# Multiple gains of spliceosomal introns in a superfamily of vertebrate protease inhibitor genes

**DOI:** 10.1186/1471-2148-9-208

**Published:** 2009-08-22

**Authors:** Hermann Ragg, Abhishek Kumar, Katharina Köster, Caterina Bentele, Yunjie Wang, Marc-André Frese, Natalie Prib, Olaf Krüger

**Affiliations:** 1 Department of Biotechnology, Faculty of Technology and Center for Biotechnology, University of Bielefeld, D-33501 Bielefeld, Germany; 2 Institute of Medical Chemistry, Center of Physiology and Pathophysiology, Medical University of Vienna, Waehringerstrasse 10, A-1090 Vienna, Austria

## Abstract

**Background:**

Intron gains reportedly are very rare during evolution of vertebrates, and the mechanisms underlying their creation are largely unknown. Previous investigations have shown that, during metazoan radiation, the exon-intron patterns of *serpin *superfamily genes were subject to massive changes, in contrast to many other genes.

**Results:**

Here we investigated intron dynamics in the *serpin *superfamily in lineages pre- and postdating the split of vertebrates. Multiple intron gains were detected in a group of ray-finned fishes, once the canonical groups of vertebrate *serpin*s had been established. In two genes, co-occurrence of non-standard introns was observed, implying that intron gains in vertebrates may even happen concomitantly or in a rapidly consecutive manner. DNA breakage/repair processes associated with genome compaction are introduced as a novel factor potentially favoring intron gain, since all non-canonical introns were found in a lineage of ray-finned fishes that experienced genomic downsizing.

**Conclusion:**

Multiple intron acquisitions were identified in *serpin *genes of a lineage of ray-finned fishes, but not in any other vertebrates, suggesting that insertion rates for introns may be episodically increased. The co-occurrence of non-standard introns within the same gene discloses the possibility that introns may be gained simultaneously. The sequences flanking the intron insertion points correspond to the proto-splice site consensus sequence MAG↑N, previously proposed to serve as intron insertion site. The association of intron gains in the *serpin *superfamily with a group of fishes that underwent genome compaction may indicate that DNA breakage/repair processes might foster intron birth.

## Background

Spliceosomal introns are key attributes of most eukaryotic genes. Their origin is still unclear, though descent from group II self-splicing introns seems to be likely [1,2]. All components essential for removal of intronic sequences, a prerequisite for maturation of most transcripts and formation of functional gene products, have been identified in basal eukaryotes [3], indicating that the ability to cope with spliceosomal introns was fully developed in the last common ancestor of eukaryotes. In addition to their obscure provenance, spliceosomal introns present another unresolved enigma. In many taxa, intron dynamics is dominated by losses, and gains of introns were often found to be rare [reviewed in ref. [2]]. In recent mass analyses of various vertebrate genomes, no intron gains were detected [4,5]. With some genes and lineages, however, intron acquisition can hardly be questioned [6-8]. Several proposals have been brought forward to explain intron birth [2,9-11], but how these sequences were actually created, is still mysterious.

The serpins are a superfamily of proteins that cover a highly divergent spectrum of functions [12,13]. The origin of these proteins, primarily encompassing inhibitors of serine proteases, but also including members with entirely other tasks, is not known. Serpins are found in all major branches of the tree of life, but they are rare in fungi, and their distribution in archaea and eubacteria is disjunct. In vertebrates, serpins participate in the control of blood coagulation, fibrinolysis, and other proteolytic pathways [12]. In both vertebrates and invertebrates, serpins are also engaged in regulating the innate immune response. An arms race between proteases of pathogens and host protease inhibitors, and *vice versa*, was proposed to foster functional diversification of serpins [14,15].

**Figure 1:**
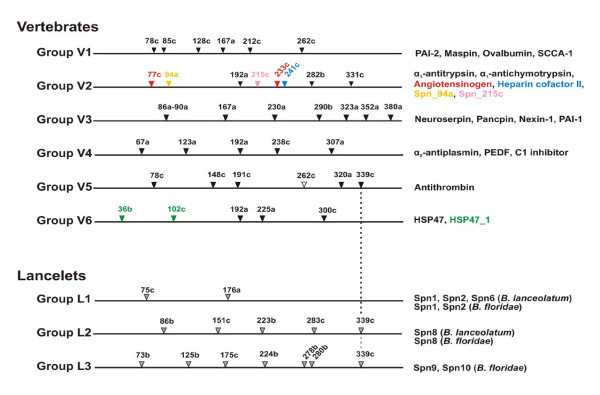
**Intron-coded classification of *serpin *genes from vertebrates and lancelets and overview on intron gain positions**. Vertebrate *serpins *are classified into six groups (V1–V6), based on group-specific sets of standard introns (black arrowheads). Characteristic representatives of each group are shown on the right. Non-canonical introns (marked in colors also used to indicate the genes concerned) are exclusively present in a lineage of ray-finned fishes, including *Oryzias latipes *(Japanese medaka), *Gasterosteus aculeatus *(stickleback), *Tetraodon nigroviridis *(green-spotted pufferfish) and *Takifugu rubripes *(Japanese pufferfish), but not in *Petromyzon marinus *and *Lampetra fluviatilis *(lampreys), *Danio rerio *(zebrafish), and tetrapods. Positions of introns (indicated on top) refer to human α_1_-antitrypsin, their phases (a-c) are given with respect to their location after the first, second or third base of the codon concerned. For comparison, *serpins *from lancelets (groups L1 to L3, intron positions indicated by grey arrowheads) have been included, demonstrating that there is little congruence concerning intron positions within the *serpin *superfamily. The intron at position 262c in group V5 (white arrowhead) is only found in fishes and was possibly lost in tetrapods. Some genes of group V1 lack the 85c intron. Some introns of L3 genes from *B. floridae *are restricted to individual members of this group (intron 280b: *Spn9*; introns 224b and 278b: *Spn10*). Due to alignment problems, the exact positions of the following introns are ambiguous: group V3, intron 86a-90a; group L1, intron 176a; group L2, intron 86b; group L3, intron 175c. Only introns mapping to the conserved serpin scaffold (amino acids 33 to 394 of human α_1_-antitrypsin) are considered.

In vertebrates, *serpin *genes are often arranged in tandem arrays and they constitute a substantial fraction of mammalian genomes. During diversification of vertebrates the superfamily has undergone considerable expansion [16,17]. Serpins are unusual compared to most other superfamilies with regards to the dynamics of gene organization. Genes from basal metazoans, such as annelids [18] or sea anemones [19], often share an intron-rich structure with their vertebrate homologues, implying that introns may be stably maintained for hundreds of millions of years. *Serpin *genes of basal metazoans, in contrast, are not generally intron-rich, and their exon-intron structures are not conserved along the lineages leading to vertebrates. Sporadic investigations of various species revealed radically different intron patterns in *serpin *genes, indicating that, during diversification of eumetazoans, massive changes in gene architectures have occurred [20,21]. The structures of *serpin *genes from various vertebrates (Figure 1), however, proved to be strongly conserved, enabling reliable, intron-coded classification of the superfamily into six groups (V1–V6). Generally, there is very little congruence between these groups concerning numbers and positions of introns. Altogether, 25 different intron positions mapping to the serpin scaffold were detected, but none of them is common to the entire superfamily [22].

Here, we investigated the structures of *serpin *genes from lineages that pre- and postdate the split of vertebrates in order to get insight into the dynamics of their introns. The data disclose that, after establishment of major groups of vertebrate *serpin *genes, multiple non-canonical introns emerged in a lineage of ray-finned fishes.

## Results

### Appearance of exon-intron patterns characteristic for vertebrate *serpins*

We first studied *serpin *genes and their cDNAs in two lancelet species, *Branchiostoma lanceolatum *(*B. lanceolatum*) and *Branchiostoma floridae *(*B. floridae*), representing a group of extant cephalochordates (phylum: Chordata). Experimental approaches disclosed several genes and cDNAs coding for serpins in *B. lanceolatum*, further superfamily members were detected by mining the genome of the closely related *B. floridae *[23]. Altogether, three types of *serpin *genes (L1–L3) with distinctly different intron patterns were observed (Figure 1; Additional file 1). Following the previous convention [22], intron positions are projected onto the sequence of the human serpin α_1_-antitrypsin. The phases of introns are indicated by the suffixes a-c, according to their location after the first, second, or third base of the codon in question. In this reference system, the two introns of L1 genes map to positions 75c and ~176a (the exact position of this intron is ambiguous, due to alignment problems). The L2 genes contain introns at positions ~86b, 151c, 223b, 283c and 339c. The L3 genes exhibit common introns at positions 73b, 125b, ~175c and 339c, however, there are also introns that are unique to individual group members (position 280b in *Bflor_Spn9*; positions 224b and 278b in *Bflor_Spn10*). Another intron in *Bflor_Spn10 *is located outside the conserved serpin scaffold (gene-specific numbering of position: 29a). There are several additional *serpin*-like sequences in the *B. floridae *genome, but it is currently not discernible whether they represent intact genes (not shown); however, it is clear that *serpin *genes from lancelets and vertebrates differ largely with respect to their exon-intron organizations. In fact, just a single intron location (position 339c) is shared. Apparently, major changes affecting the exon-intron patterns of *serpin *genes have occurred since the cephalochordate/vertebrate split.

Having established lancelets as appropriate outgroup for evaluating evolution of *serpins *in vertebrates, we turned to lampreys, a group of basal, jawless fishes. Lampreys, in sharp contrast with lancelets, depict at least four of the six canonical groups of vertebrate *serpins*. A survey of cDNA and genomic sequences from *Lampetra fluviatilis *(*L. fluviatilis*; European river lamprey) and from *Petromyzon marinus *(*P. marinus*; sea lamprey) revealed representatives of groups V2, V4 and V6 (Additional file 2). We also infer that intact members of group V1 exist as indicated by the isolation of a corresponding full-length cDNA from *L. fluviatilis*. The associated gene contains all introns characteristic for group V1 with the exception of the 78c intron that, presumably due to its large size, remained undetected (unpublished results). Members of groups V3 and V5 were not identified.

**Figure 2:**
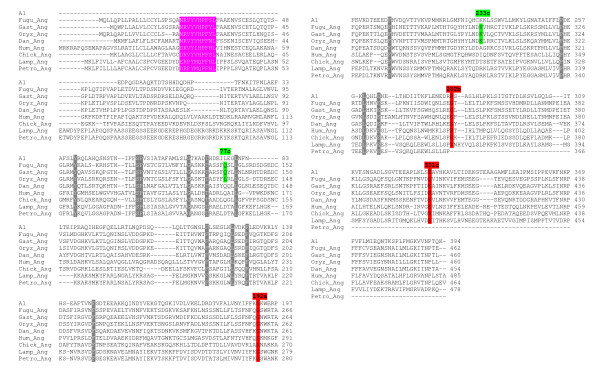
**Alignment of angiotensinogen sequences and intron location analysis**. Angiotensinogen sequences were aligned together with mature human α_1_-antitrypsin (A1) serving as reference protein. The following color code is used to characterize introns: red, standard introns; green, non-canonical introns exclusively present in *Oryzias latipes*, *Gasterosteus aculeatus *and *Takifugu rubripes *(Fugu), but not in lampreys (*Petromyzon marinus*, *Lampetra fluviatilis*), tetrapods (human, chicken) and *Danio rerio*. Positions and phases (a-c) of introns are depicted above the alignment and refer to human α_1_-antitrypsin. The angiotensin signature sequence is reproduced in white on pink background. Residues conserved in all sequences are printed in white on grey background.

Inspection of lamprey serpin sequences disclosed the presence of angiotensinogen and heparin cofactor II (HCII), two prominent members of group V2. All known angiotensinogen proteins depict a conserved decapeptide sequence close to the N-terminus that, after controlled enzymatic cleavage, gives rise to formation of peptides (angiotensin I-IV) involved in blood pressure regulation and other important physiological processes [24]. Clearly, such a sequence is also present in angiotensinogen orthologues from *L. fluviatilis *and *P. marinus *(Figure 2). The NVIYFKG signature (positions 268–274 in *L. fluviatilis*), among other features, definitely reveals this protein as member of the serpin superfamily.

**Figure 3:**
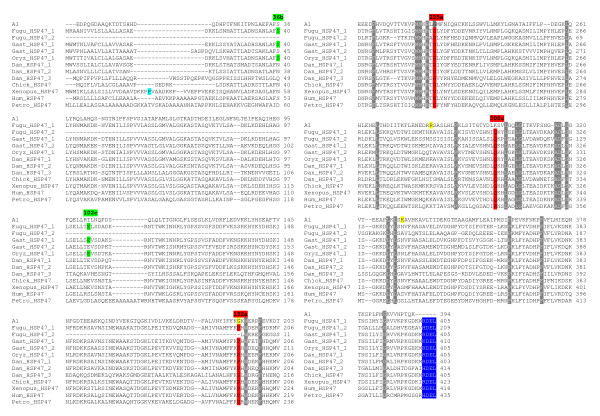
**Intron gain in group V6 during diversification of ray-finned fishes**. Ray-finned fishes contain up to three *HSP47*-related genes basically sharing the intron pattern of group V6 (intron positions marked in red). Non-canonical introns (positions marked in green) are exclusively found in some *HSP47*-related genes *from Oryzias latipes*, *Gasterosteus aculeatus *and *Takifugu rubripes*, but not in *Danio rerio *or other members of group V6. An intron located outside the serpin scaffold of *X. tropicalis HSP47 *is depicted in turquoise. Intron positions of human α_1_-antitrypsin (A1, group V2) are shown in yellow. The characteristic ER retention signal present at the C-terminus of all members of group V6 is printed in white on blue background. Sequence alignments and intron mapping were accomplished as described in the legend to Figure 2.

HCII, a serpin well known from various tetrapods, is a potent thrombin inhibitor in the presence of glycosaminoglycans (GAGs). Characteristic features of all HCII sequences are the highly conserved Arg/Lys-rich helix D that is involved in GAG binding and the acidic N-terminal extension that mediates GAG accelerated thrombin inhibition [25,26]. These features are also found in lamprey HCII (Additional file 1). The genes coding for angiotensinogen and HCII from lampreys each depict introns that interrupt the serpin scaffold at positions 192a, 282b, and 331c (standard repertoire of group V2; positions of group-specific standard introns are marked in red in all figures showing alignments). Beyond that, there are additional introns mapping to the N-terminus of HCII from *P. marinus *(see below). We also recognized a lamprey *serpin *exhibiting the exon-intron pattern of group V6 (introns at positions 192a, 225a, and 300c; Figure 3) as *HSP47 *orthologue. HSP47, a non-inhibitory serpin, is a specialized ER residing chaperone involved in folding and transport of procollagens [13,27]. A hallmark of all HSP47 proteins is the C-terminal ER retention/retrieval signal (HDEL/KDEL/RDEL). We conclude that angiotensinogen, HCII, and HSP47 are distinct members of the serpin superfamily that appeared early during vertebrate evolution. These proteins have persisted since at least 360 million years, assuming that the morphological concordance between a fossil lamprey from the Devonian period [28] and its present-day relatives is reflected on the molecular level.

### *Serpin *genes with non-standard exon-intron patterns

After curtailing the emergence of major groups of vertebrate *serpin *genes, the dynamics of their exon-intron patterns was analyzed. The list of species investigated included: man, chicken (*Gallus gallus*), the clawed frog (*Xenopus tropicalis; X. tropicalis*), and several fishes including *Danio rerio *(*D. rerio*), *Oryzias latipes *(*O. latipes*),* Gasterosteus aculeatus *(*G. aculeatus*), *Tetraodon nigroviridis *(*T. nigroviridis*), and *Takifugu rubripes *(*T. rubripes*). Several *serpin *genes, though clearly members of one of the six canonical groups, were found to deflect from the standard organizations. Like their counterparts in lampreys, *angiotensinogen *genes of tetrapods and *D. rerio *exhibit the canonical gene architecture of group V2, the *T. rubripes *orthologue, however, contains two extra introns that split the exonic sequences at positions 77c and 233c, respectively (Figure 2; non-standard introns are marked in green). These surplus introns are also present in the orthologues of *O. latipes *and *G. aculeatus*, but they are not found in any other vertebrate *serpins*. In the current release of the *T. nigroviridis *genome, the *angiotensinogen *gene is not represented. EST data revealed (Additional file 2) that, in *G. aculeatus*, the extra introns are indeed spliced out, rejecting the argument that they are artifacts.

The *HCII *genes of mammals [29,30], chicken, *X. tropicalis *and *D. rerio *uniformly depict the conserved intron pattern of group V2. Deviations from the standard structure, however, were observed in lampreys and in several fishes. The *HCII *orthologues of *O. latipes*, *G. aculeatus, T. rubripes *and *T. nigroviridis *each contain a non-standard intron mapping to position 241c (Additional file 1). EST analyses confirmed expression and correct splicing of the *HCII *transcript in *O. latipes *(Additional file 2). To our knowledge, there are no other vertebrate *serpins *possessing an intron at this position. A gain that occurred after the split of the *D. rerio *lineage from the other ray-finned fishes thus may explain appearance of this intron. Another extra intron in the *HCII *genes of pufferfishes maps to the inhibitor's N-terminal tail (gene-specific numbering of positions: 85b and 87b for *T. rubripes *and *T. nigroviridis*, respectively). Though also found in *O. latipes *and *G. aculeatus*, this intron here is not considered any further, due to its location outside the serpin scaffold. Examination of lamprey *HCII *also revealed unique introns. The 83c intron (α_1_-antitrypsin numbering) is embedded in a well-conserved region; its origin, however, is difficult to evaluate. The others (correctly predicted?) map to the N-terminal extension (gene-specific numbering of positions: 38b and 118c).

Database searches also disclosed members of group V2, dubbed *Spn_94a*, with a surplus intron at position 94a (Additional file 1). In *O. latipes*, *G. aculeatus*, *T. rubripes *and *T. nigroviridis*, these genes are flanked by a conserved set of markers (Additional file 3). The imbedded *serpin *genes thus are derived from a common ancestor. Position 94a was previously not known to harbor introns in vertebrate *serpins*. Inspection of chromosomal gene order revealed that *D. rerio *also contains *Spn_94a*; the extra intron, however, is missing, suggesting that it was gained after divergence of the *D. rerio *lineage. In pufferfishes, two further members of group V2 with an extra intron, located at position 215c, were identified (*Spn_215c *from *T. rubripes *and *T. nigroviridis*, respectively). The origin of these genes is unclear; the unique surplus intron suggests that they share a common ancestor (Additional file 1).

**Figure 4:**
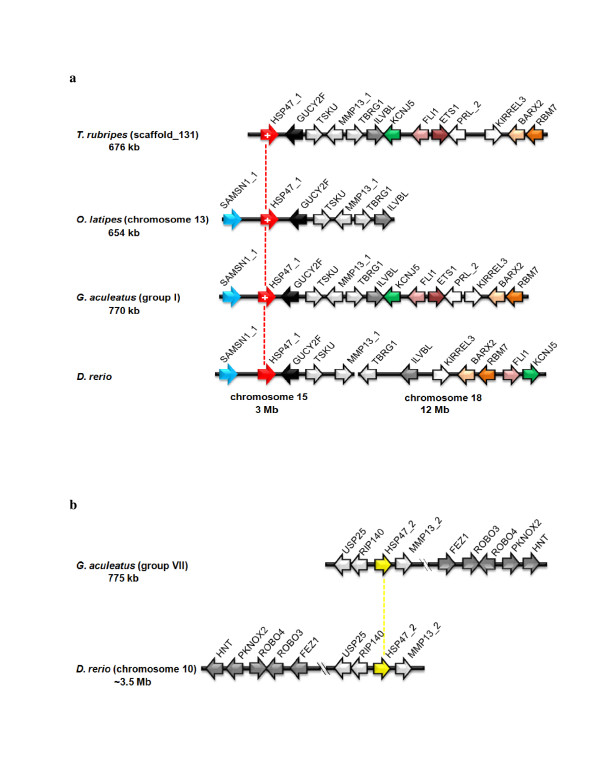
**Intron gain in group V6 is restriced to *HSP47_1 *orthologues**. Gene orders reveal that, with the exception of *Danio rerio*, non-canonical introns (symbolized by a plus sign) occur exclusively in *HSP47_1 *orthologues (a), but not in the paralogous *HSP47_2 *genes (b) or in *HSP47_3 *(only known from *Danio rerio*; not shown). Intron acquisition in *HSP47_1 *orthologues occurred during radiation of ray-finned fishes following divergence of the *Danio rerio *lineage, as suggested by lack of the extra introns in all other members of group V6, including tetrapods and lampreys.

In most mammals, chicken, *X. tropicalis *and in *P. marinus*, group V6 encompasses a single member, *HSP47*, uniformly depicting introns at positions 192a, 225a and 300c. In *D. rerio*, however, there are three *HSP47-*related genes (*Dan_HSP47_1*, *Dan_HSP47_2*, and *Dan_HSP47_3*), all of which are equipped with the standard set of introns (Figure 3). Neighbor-joining analyses of HSP47 proteins and reference serpins from groups V1–V5 confirmed phylogenetic clustering of group V6 genes from *D. rerio *(not shown), which probably arose as a consequence of genome duplication events in the stem lineage of ray-finned fishes [31,32]. In the other actinopterygians investigated, the phylogenetic history of group V6 is less clear, partly due to the varying status of the still ongoing genome sequencing projects. Orthologues of *Dan_HSP47_1*, as indicated by the conserved gene order (Figure 4a) were detected in *G. aculeatus*, *O. latipes *and *T. rubripes *(dubbed *Gast_HSP47_1, Oryz_HSP47_1 *and *Fugu_HSP47_1*, respectively). In *G. aculeatus*, a second intact *HSP47 *homologue, *Gast_HSP47_2*, was identified. *Dan_HSP47_2 *and *Gast_HSP47_2 *are orthologous to each other, since they share a set of flanking markers (Figure 4b) that proved to be reciprocal best hits in BLAST searches (not shown). Currently it is not clear, whether some further *HSP47*-related sequences present in the genomes of *T. rubripes *and *O. latipes *represent incompletely annotated or defective genes. The only *HSP47*-related sequence detected in the *T. nigroviridis *genome is incomplete.

The intron patterns of *HSP47 *homologues uncover telling insight into the evolution of group V6 in fishes (Figure 3). *Dan_HSP47_1*, *Dan_HSP47_2*, *Dan_HSP47_3*, and *HSP47 *from *P. marinus *and tetrapods, respectively, all depict the standard intron repertoire. *Gast_HSP47_1*, *Oryz_HSP47_1 *and *Fugu_HSP47_1*, however, contain two additional introns (positions 36b and 102c, respectively); *Gast_HSP47_2*, in contrast, merely possesses the default introns. These findings suggest that the novel introns, which are restricted to *HSP47_1 *orthologues, were acquired by group V6 during evolution of ray-finned fishes after divergence of the *D. rerio *lineage, though an intron loss scenario cannot be excluded. Two intron gains or a single, coupled intron gain event are/is sufficient to explain the exon-intron patterns found in group V6; the intron removal scenario, in contrast, requires multiple intron loss events. If such losses had occurred, they must have affected several taxa, including lampreys, tetrapods and fishes. Moreover, this scenario also demands that the same two introns (positions 36b and 102c) were always deleted in parallel, while all other introns were unaffected. The parallel emergence of introns at positions 77c and 233c in *angiotensinogen *genes (Figure 2) provides further support for the intron gain presumption.

In contrast with several intron gains, we detected a single case of probable intron loss during evolution of vertebrate *serpins*. Antithrombin (AT), the only member of group V5, is a potent thrombin inhibitor in the presence of heparin. Among other characteristic features, AT orthologues are easily discernible through a highly conserved sequence centering around helix D (Additional file 1) that constitutes a major part of the heparin binding region. The *AT *genes of tetrapods have introns interrupting the serpin scaffold at positions 78c, 148c, 191c, 320a and 339c (Figure 1). The orthologues from fishes, however, exhibit an additional intron at position 262c (Additional file 1). This intron is also present in *D. rerio *and in the currently incompletely annotated *AT *gene of *T. nigroviridis *(not shown). Intron 262c is a standard attribute of group V1 that is believed to share a common ancestor with group V5 [22]. We therefore suspect that this intron was lost in *AT *genes of tetrapods. We were not able to identify this gene in *P. marinus*; further tracing of the 262c intron was therefore not possible.

In *serpins *from groups V3 and V4, deviations from the standard structures were not observed (not shown). All genes from group V3 analyzed featured introns at positions ~86a-90a, 167a, 230a, 290b, 323a, 352a, and 380a. The intron pattern characteristic of group V4 (positions 67a, 123a, 192a, 238c, 307a) was also conserved.

### Features of novel introns

Table 1 summarizes some properties of the non-canonical introns identified. Their sizes vary from 68 to 178 bp (for sequences see Additional file 4), about the average range found for introns of *T. rubripes *[33]. All novel introns are bounded by canonical GT-AG splice signals, the GC content extends from 26.8 to 55.9%. Five out of seven introns interrupt the open reading frame between codons (phase c) and one each after the first or the second base of the codon, respectively. No similarities to known complex repetitive elements were detected. In five out of seven cases the novel introns are embedded in a fairly well conserved sequence environment with no insertions or deletions. In contrast, the sequences flanking the 94a intron of *Spn_94a *orthologues cannot be aligned without introducing gaps (Additional file 1). These considerations are not applicable to the 215c intron of *Spn_215c*, since the ancestor is unknown. Intron gain was proposed to occur at preferred locations (consensus sequence: C/AAG↑N), referred to as proto-splice sites [34,35]. We examined the sequences enclosing the insertion points of non-standard introns in *serpins *of ray-finned fishes (Table 1). Generally, the sequences flanking the intron insertion points are concordant with the proto-splice sites proposed. We note that a relatively high fraction of bases immediately adjacent to the splice acceptor site are pyrimidine residues.

**Table 1:** Sequences flanking the insertion points of novel introns in vertebrate *serpin *genes.

Species	Gene	Intron	Flanking sequences
*T. rubripes*	*Angiotensinogen*	77c (75)	CCAG↑TCTC
*G. aculeatus*	*Angiotensinogen*	77c (140)	CCAG↑TACC
*O. latipes*	*Angiotensinogen*	77c (82)	TCTG↑CGTC
			
*T. rubripes*	*Angiotensinogen*	233c (80)	TAAG↑GTTC
*G. aculeatus*	*Angiotensinogen*	233c (112)	TAAG↑GTAC
*O. latipes*	*Angiotensinogen*	233c (80)	TAAG↑TTGA
			
*T. rubripes*	*HCII*	241c (75)	ACAG↑CTCC
*T. nigroviridis*	*HCII*	241c (70)	ACAG↑CTCC
*G. aculeatus*	*HCII*	241c (82)	ACAG↑CTCC
*O. latipes*	*HCII*	241c (98)	ACAG↑CTCC
			
*T. rubripes*	*HSP47_1*	36b (178)	TCAG↑CCTC
*G. aculeatus*	*HSP47_1*	36b (141)	TCAG↑CCTC
*O. latipes*	*HSP47_1*	36b (100)	TTAG↑CCTT
			
*T. rubripes*	*HSP47_1*	102c (88)	TGAG↑TTGA
*G. aculeatus*	*HSP47_1*	102c (123)	CGAG↑GTGA
*O. latipes*	*HSP47_1*	102c (97)	TGAA↑GTGA
			
*T. rubripes*	*Spn_94a*	94a (68)	CCAG↑AGCT
*T. nigroviridis*	*Spn_94a*	94a (68)	CCAG↑ATCT
*G. aculeatus*	*Spn_94a*	94a (74)	CCAG↑ATCT
*O. latipes*	*Spn_94a*	94a (111)	CCAG↑ATCT
			
*T. rubripes*	*Spn_215c*	215c (76)	CAAG↑GTTC
*T. nigroviridis*	*Spn_215c*	215c (68)	CAAG↑GTCC

Arrows indicate the intron insertion points. Intron sizes are given in brackets.

## Discussion

Intron gain has been reported to occur very rarely in many metazoan lineages, including mammals and other vertebrates [4,5]. To our surprise we disclosed multiple newly acquired introns by probing a vertebrate protein superfamily, the serpins, while a single intron was presumably lost. The most clear-cut example for intron gain is *angiotensinogen *that, in lampreys, tetrapods and *D. rerio*, depicts the typical exon/intron pattern of group V2. The novel introns at positions 77c and 233c, like all other non-standard introns identified, are exclusively found in a group of ray-finned fishes that emerged after the split of the *D. rerio *lineage. None of the novel intron positions is found in any paralogues of group V2 or in any other vertebrate *serpins *known. Thus, from a parsimony standpoint, the view that these introns were acquired *de novo *is more likely than the alternative possibility that these introns were inherited from a common ancestor. The novel introns were apparently not acquired at the expense of adjacent introns, as concomitant loss of such sequences was not observed. Since there are no reports of intron gain in *serpins *of other vertebrates, our findings may reflect an episode of enhanced intron acquisition that happened during radiation of ray-finned fishes. Several of the few other well-documented intron gains also occurred during diversification of these fishes [36-39].

**Figure 5:**
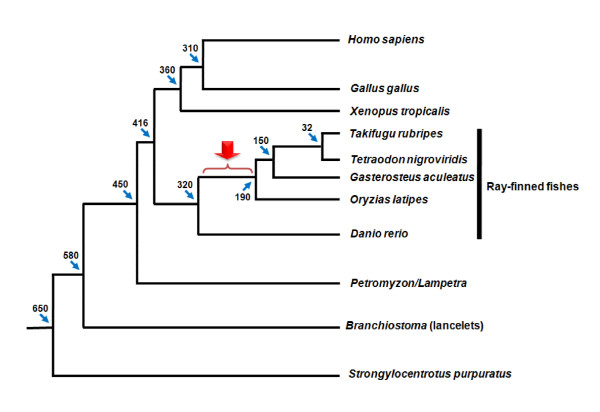
**Phylogenetic tree of vertebrates emphasizing timescale and lineages displaying intron gain in *serpin *genes**. The estimated divergence times (in mya), taken from ref. [40], are marked with blue arrows. The time interval of intron gains in ray-finned fishes is indicated (red arrow).

*HSP47 *genes are especially informative concerning both the time period and the processes possibly associated with intron birth. During evolution of ray-finned fishes, group V6 was split into three lineages, probably a consequence of whole genome and/or large fragment duplications. The extra introns at positions 36b and 102c, however, were acquired only by *HSP47_1 *orthologues after divergence of the *D. rerio *lineage. *HSP47_2 *from *G. aculeatus*, in contrast, depicts the standard intron pattern, just as *HSP47_1*, *HSP47_2 *and *HSP47_3 *from *D. rerio *and the members of group V6 from lampreys and tetrapods. These findings indicate that intron gains were not associated with the fish-specific genome duplication events, they rather support the view that co-existence of paralogues may favor maintenance of introns once gained. Phylogenetic data [40] locate birth of all new introns in *serpins *to a time period about 320-190 mya (Figure 5).

Except for *Spn_94a*, the insertion points of novel introns are exact, as no deletions or insertions at the flanking sequences are evident. Many serpins tolerate indels without functional impairment, especially in loops connecting α-helices and β-strands, a noteworthy aspect that should be kept in mind in the discussion of intron gain mechanisms. Inspection of nucleotide sequences in the neighborhood of novel introns revealed compliance with the proto-splice site sequences proposed [34,35]. A relatively high fraction of bases immediately adjacent to the splice acceptor site are pyrimidine residues, possibly biased due to the limited number of samples analyzed or due to subsequent selection processes. Alternatively, this finding may indicate that the specifications required at the 3'-side of intron insertion sites are low.

Several mechanisms have been claimed to be responsible for birth of introns [2,41,42]. Key to most of the proposals is duplication events operating at some stage. A popular mechanism thought to mediate intron gain involves transposons. A distinguishing feature of fish genomes is their diversity of retrotransposable elements, notably retrotransposons, some of which were active in recent times [33,43,44]. However, we could not detect any sequences in non-standard introns sharing similarity with known repetitive elements, participation of duplication-dependent transposons with intron gain therefore remains elusive. Preferential loss of such elements from newly acquired introns, however, cannot be excluded. In two recently described cases of intron acquisition, similarity to other genomic sequences was also not observed [7].

We consider that various processes might be responsible for intron birth, not necessarily related with the events responsible for their primordial emergence. Excision of introns, probably created by expansion of simple repeats or complex repetitive elements [36,45] or generated by intronization of exon sequences [41], demonstrates that the spliceosome can do its job as long as the essential splice signals are present, irrespective of how the intron was created. Since we were not able to find support in favor of currently discussed mechanisms of intron gain (reviewed in [2,9-11]), we consider that introns might also be created by other means. After the fish-specific whole genome duplication, compaction processes resulted in reduction of genome sizes in many actinopterygians [33,46-48]. Deprivation of genomic DNA not only entailed loss of complete genes, but also affected intergenic and intronic sequences. Intron sizes, for instance, are considerably larger in *D. rerio *than in pufferfishes. It remains to be investigated, whether all these sequences have gone without leaving any traces behind. Loss of genomic sequences is inevitably associated with DNA breakage, requiring repair and recombination. The involvement of such processes in intron acquisition should therefore be considered; however, like for the other intron gain mechanisms suggested, conclusive evidence of a causal relationship between DNA breakage/repair and de novo intron formation is lacking as yet.

Genome-scale or local amplification of genes might conceivably favor gain and maintenance of novel introns, since unaffected copies are left in the genome. The appearance of novel introns in the *serpin *superfamily, however, was apparently not associated with the fish-specific genome duplication that preceded radiation of ray-finned fishes [32]. In current phylogenetic scenarios, divergence of *D. rerio*, which lacks all of the non-canonical introns, antedates the split of the lineage that experienced intron gains. Retention of introns once acquired might indeed be favored by the co-existence of paralogues, relative frequencies of intron gains in single versus multi-copy gene families, however, are discussed controversially [49,50].

## Conclusion

By a comprehensive analysis of lineages pre- and postdating the split of vertebrates, the founding period for major groups of vertebrate *serpins *was ascertained. Following establishment of the canonical exon-intron patterns before or close to diversification of lampreys, a lineage of ray-finned fishes is shown to have experienced multiple intron gains. Remarkably, in two genes concomitant appearance of non-canonical introns is observed, suggesting that intron gains may even happen in parallel or in a rapidly consecutive manner. These data strongly suggest that intron acquisition occurs in at least some vertebrate taxa. The observation that all intron gain events were found in a lineage of ray-finned fishes that underwent genome compaction, leads us to assume that DNA breakage/repair processes may enable or facilitate intron acquisition. Angiotensinogen, HCII, and HSP47 were identified as ancient members of the serpin superfamily in vertebrates.

## Methods

### Materials

Adult lancelets (*B. lanceolatum*) were purchased from the Alfred-Wegener-Institut für Polar- und Meeresforschung, Helgoland, Germany. European river lampreys (*L. fluviatilis*) were obtained from the Bundesforschungsanstalt für Fischerei, Hamburg, Germany.

### Cloning and sequencing of serpin cDNAs and genes

The isolation of poly(A)-RNA and genomic DNA, the synthesis of cDNA, PCR amplification of serpin cDNA fragments using various sets of degenerate primers, and cloning of 5'- and 3'-cDNA ends followed published procedures [20,51]. DNA sequences (Additional file 2) have been deposited in the DDBJ/EMBL/GenBank database.

### Sequence data analysis and intron annotation

Genomic data for *serpins *from human, chicken [52], *X. tropicalis *[53],* D. rerio *[54],* G. aculeatus *[55], *O. latipes *[56], *T. rubripes *[33] and *T. nigroviridis *[31] were extracted from the Ensembl genome browser, release 51 [57], or in the case of *P. marinus *[58], from PreEnsembl (). Sequences from the *B. floridae *genome were gathered from the JGI genome browser (). EST and cDNA data mining included searches in the NCBI trace archive () and in the UCSC genome browser [59], applying the BLAST algorithm. Some gene models were refined using EST data (Additional file 2).

All intron positions predicted by gene models were examined visually, corrected and amended manually, if necessary. Whenever cDNA or EST sequences were available, intron positions were checked by means of GENEWISE [60]. Protein sequences were aligned with CLUSTALW [61] with some manual improvements. Intron positions were projected onto the sequence of mature human α_1_-antitrypsin as described [22]. All intron locations allude to the reference protein, unless stated otherwise. Only introns mapping to the conserved serpin scaffold (*i.e*. positions 33 to 394 of human α_1_-antitrypsin) were considered.

Sequences of non-canonical introns were searched for repetitive elements with the RepeatMasker package (version 3.2.6; ()) and with RepBase Censor () [62] using default settings.

Phylogenetic analysis was performed using the Neighbor-Joining method [63] conducted in MEGA4 [64]. All positions containing gaps and missing data were eliminated from the dataset (complete deletion option). There were a total of 340 positions in the final dataset.

## Authors' contributions

HR designed the study, performed part of data analyses, and wrote the paper. AK accomplished data analyses. KK, YW, CB, MAF, NP and OK generated data and contributed to data evaluation.

## Supplementary Material

Additional file 1 **Mapping of intron positions to aligned serpin sequences**. Figure depicting intron positions of *serpin *genes mapped onto the aligned amino acid sequences. Click here for file

Additional file 2 **List of *serpin *genes analyzed in this study and their accession numbers**. Table listing accession numbers of genes, cDNAs and ESTs investigated in this study. Click here for file

Additional file 3 **Chromosomal gene order reveals orthology of *Spn_94a *genes**. Figure showing chromosomal synteny of *Spn_94a *genes. Click here for file

Additional file 4 **Sequences of non-canonical introns from vertebrate *serpin *genes**. Figure depicting the sequences of non-standard introns. Click here for file
