# Supplementary material for: Multiple gains of spliceosomal introns in a superfamily of vertebrate protease inhibitor genes
Source: BMC Evol Biol. 2009 Aug 22;9:208. doi: 10.1186/1471-2148-9-208 (PMC2746811; doi:10.1186/1471-2148-9-208)
Supplement: Additional file 1 — Mapping of intron positions to aligned serpin sequences. Figure depicting intron positions of serpin genes mapped onto the aligned amino acid sequences. [file 1471-2148-9-208-S1.pdf]

Additional file 1. Mapping of intron positions to aligned serpin sequences.

The positions of introns are indicated on top (α_1-antitrypsin numbering system).

A1: mature human α_1-antitrypsin (reference sequence)

List of serpin sequences aligned

1) Serpins of the genus *Branchiostoma*

2) HCII sequences

3) Spn_94a sequences

4) Spn_215c sequences

5) AT sequences

1) Serpins of the genus *Branchiostoma*

Blanc: serpins from *Branchiostoma lanceolatum*

Bflor: serpins from *Branchiostoma floridae*

turquoise: intron position outside conserved serpin domain

pink: intron positions of L1 serpins

yellow: intron positions of L2 serpins

green: intron positions of L3 serpins

red: intron positions of human α_1-antitrypsin

```
A1                   -----------EDPQGDAAQKTDTSHHDQDHP------TFNKITPNLAEFAFSLYRQLAH 43
Blanc_Spn1           ---------MPGHSYSLFGLLVAMATLGSSAS-----QESTPLADINSEFALELYKTLHK 46
Blanc_Spn6           ---------MPGHSYCLFGLLVAMATLGSSAS-----QESTPLADVNAEFALELYKTLHK 46
Bflor_Spn1           ---------MLGHSCCLFGLLVAMTTMRSSTS-----QESTPLADINSEFALELYKALHK 46
Blanc_Spn2           ---------MRSTYCLFIGLLVAMATAENSAP-----QEPTSLADVNSEFALELYKALHK 46
Bflor_Spn2           ---------MLCPHYWLLGLLVAIATTENAFP-----QEPTSLADINSEFALELYKVLHK 46
Blanc_Spn8           ------------------------------------------------------------
Bflor_Spn8           ------------------------------------------------------------
Bflor_Spn9           MSNFYSVYLLPECITFIYLHQRCVLAFLEPIP----VMSEDEVAEANSAFALSLYRQLSQ 56
Bflor_Spn10          MGGKKKIKNRRKPPARLRGRERTVSASTGVTTSWEAKSSLSSLVEANSAFALGLFRRLCD 60

                                                  73b75c      ~86b-88b
A1                  QSNSTNIFFSPVSIATAFAMLSLGTKADTHDEILEGLNFNLTEIPEAQIHEGFQELLRTL 103
Blanc_Spn1          DHP-ENIFFSPFSISTCLAMAYLGARNDTAQQMSRVLRFHKMD--ASDLHVLFHDLLTQL 103
Blanc_Spn6          DHP-ENIFFSPFSISTCLAMTYLGARNDTAQQMSRVLRFHKMD--ASDLHMLFHDLLTQL 103
Bflor_Spn1          DHP-ENIFFSPFSISTCLAMTYLGARNDTAQQMSRVLRFHKMD--ASDLHMLFHDLLTQL 103
Blanc_Spn2          DHP-ENIFFSPFSISTCLAMTYLGARNETAQQMSHVLRFNKVN--QTDFHERFHHLLTQV 103
Bflor_Spn2          DHP-ENIFFSPFSISTCLAMTYLGARNDTAQQMSRVLRFNKLN--QTDFHDRFRDLLAQL 103
```

```
Blanc_Spn8      ------MFVSPLSISTALAMTYLAAKGKTAEQMGKTMHFDDLS--ELTLHKTFAELTETT 52
Bflor_Spn8      ------MFVSPLSISTALAMTYLAAKGKTAEQMGKTMHFDDLS--ELTLHKTFAKLTETT 52
Bflor_Spn9      RTD-GNIFFSPYSISAALAMTYMGARHTTAAQMAEVLHLT-----EGDFHQAFSNLS--R 108
Bflor_Spn10     STD-GNIVFSPLSISAAMAMTYIGARGNTRYQMERILRFHYFQN-EDDLHSTFSAIEDVI 118
                      :..** **:::.:**  :.::  *  :: . :.:        :*  *  :

                                     125b                        151c
A1              NQPDS--QLQLTTGNGLFLSEGLKLVDKFLEDVKKLYHSEAFTVNF-GDTEEAKKQINDY 160
Blanc_Spn1      HHSDR--PYTLKTANRLFGQNSFEFSQKFLDETSRHYRAQLAPVDFSGNTEGARQTINSW 161
Blanc_Spn6      HHPDR--PYTLKTANRLFGQNSFEFAQKFLDETSRHYRAQLAPVDFSGNTEGARQTINSW 161
Bflor_Spn1      HHSDR--PYILKTANRLFGQNSFEFVQKFLAETSRHYRAQLAPVDFHGNTEGARQTINSW 161
Blanc_Spn2      HHPDR--LYTLKTANRLFGQNSFKFGQKFLDETSRNYGAELAPLDFRGNTEGARQTINNW 161
Bflor_Spn2      HHSDR--PYILKTANRLFGQNSFKFVQKFLDETSRHYGADLAPVDFHGNSEGARQTINSW 161
Blanc_Spn8      STNTT--AYTLSMANRLFVQEDFNLLQTYVDGMKQHYGAEAGRVNF-GDTKVASDMINNW 109
Bflor_Spn8      STNMT--SYTLSMANRLFVQEDFDVLQSYIDGMKQHYGAEVGRVDF-GDSKVASDMINNW 109
Bflor_Spn9      TMFGNLKKHTLVEANKLFGQQGMKLEDDFLSGTSRYYNARMEKVDFFDEER-SRSRINSW 167
Bflor_Spn10     STSGREADYTFVQANRLFGQAGMSFRHDFLMDTSRHYHSSLATVEFSDEEM-ARLAINSW 177
                          :  .* **  . .:.. . ::   .: * :    ::* .:   :   **.:

                             175c
                             ~176a              192a
A1              VEKGTQGKIVDLVKE--LDRDTVFALVNYIFFKGKWERPFEVKDTEEEDFHVDQVTTVKV 218
Blanc_Spn1      VEEQTENKIQDLLAPGTVTPATMLVLVNAIYFKGSWERKFEESRTRLGTFHISRDEKVEV 221
Blanc_Spn6      VEEQTENKIQDLLAPGTVTPATMLVLVNAIYFKGSWERKFEESRTRLGTFHVSRDEKVEV 221
Bflor_Spn1      VEEQTENKIQDLLAPGTVTPSTMLVLVNAIYFKGSWESKFEESRTRLGTFHISRDEKVEV 221
Blanc_Spn2      VEEQTDNRIQEIMEPGFLSPETVLVLVNAIYFKGSWESPFYTSDTMLNSFHVNPEEDVQV 221
Bflor_Spn2      VEEQTENKIQDIMAPGSVSPETLLVLVNAIYFKGKWESQFYSSDTMLRPFHVNHEEKVQV 221
Blanc_Spn8      VEEKTRQKIQDLISEDMLSELTRLVLINAIYFKAKWNDEFDPFDTQDRPFFKTEEDSVDV 169
Bflor_Spn8      VEEKTQQKIQDLISEDMLNDLTRLVLVNALYFKAKWDNEFNPFDTDDRPFFRTEEDSVDV 169
Bflor_Spn9      VSTQTKRKINDLIPKDVLNALTRLVLVNAVYFKGTWQTQFDPRETYDRKFFASSGNHVTT 227
Bflor_Spn10     VAGRTGGKVKGVIPQGLLKPLTKLVLVNAVYFAGKWRTEFDPQLINMADFFIGPERAVKV 237
                *   *  ::  ::    :   * :.*:* ::* ..*   *         *.       * .

                  223b
                   224b
A1              PMMKRLGMFNIQHCKKLSSWVLLMKYLGNATAIFFLPDEGK--LQHLENELTHDIITKFL 276
Blanc_Spn1      PMMHQQGRFKLAYDEDLNCQILEMPYQGKHLSMLLVLPEKMDALSTIETSLTPDILRRWQ 281
Blanc_Spn6      PMMHQQGRFKLAYDEDLNCQILEMPYQGKHLSMLFVLPEKMDALSTIETSLTPDLLRRWQ 281
Bflor_Spn1      PMMHQQGRFKLAYDEDLNCQILEMPYRGKHLSMVVVLPDKMDDLSAIETSLTPDLLRHWR 281
Blanc_Spn2      PMMYQDGMFKFGRDENLNCQILEMPYKGKHLSMVLVLPDKIDGLNAIETSLTPELLNRWQ 281
Bflor_Spn2      PMMYQDGIFKLGRDDDLNCSTLEIPYKGKHLSMMVVLPDEIGGLKTIETSLTPEVLQKWQ 281
Blanc_Spn8      PMMHREGHFNLVIDPEVGCSVLELPYKEKDLSMLVIVPTEKEGLGQVEDQVTMETLQGWN 229
Bflor_Spn8      PMMHRSGNHHILFDPEVGCSVLELPYKQRDLSMLVIVPTEKEGLRQVEDKITMDTLRGWR 229
Bflor_Spn9      PTMHQRGKFRMADLPNLRCRMLELPYAGDELAMFVILPKQMFGLKDVEAVLTSEALLDAT 287
Bflor_Spn10     PIMQLSGEFNVTEDPSLDCAVVELPYSGNEIVMDIVLPNQRDGLERLQGQLTRRALNRIF 297
                * *   * ...    .: .  : : *      : .:       *  ::  :*   :

                278b282b
                  280b283c                                              331c
A1              ENEDRRSA---SLHLPKLSITGTYDLKSVLGQLGITKVFS-NGADLSGVTEEAPLKLSKA 332
Blanc_Spn1      KSMDEVST---MVQIPKFKLVHDFVLNEKLADMGMTDLFSMADADLSGITGSRDLHVSQV 338
Blanc_Spn6      KSLDEVST---MVQIPKFKLVHDFVLNEKLADMGMTDLFSMADADLSGITGSRNLHVSQV 338
Bflor_Spn1      KSMSEEST---MVQIPKFKVEQDFLLKEKLAEMGMTDLFSMADADLSGITGSRDLHVSHV 338
Blanc_Spn2      NSMVKEDV---GILIPKFKLVQDFGLSEKLSEMGMPDLFG-TNVDLSGMTGSRDLHVDAL 337
Bflor_Spn2      KSMVKEDV---GILMPKFKLEQDFGLSEKLSEMGMPDLFG-TDADLSGMTGSRDLHVDAL 337
Blanc_Spn8      NKMVNKLA---FVYLPKFKLEYAVTLTDHLKQMGMEDLFDATLADLSGLTGSKDLYVSDV 286
Bflor_Spn8      NALNDTFS---LVYLPKFKLEYSVSLTEHLKQMGMEDLFDSRLADLSGLTGSRDLHVSQV 286
Bflor_Spn9      RSKSLQEVRSLDVALPKFRLTHALSLKNQLTALGMTDLFSMETADLSGVTGEKGLHVSEV 347
Bflor_Spn10     RRYLPLEG---SVLLPKFHLTEEFSLKAQLTAMGMDDLFSQNRADLSGMTGQPGMHVSDA 354
                .           : :**: :     *.  *  :*: .:*.   .****:*  .  : :.

                     339c
                     339c
A1              VHKAVLTIDEKGTEAAGAMFLEAIPMSIP------PEVKFNKPFVFLMIEQNTKSPLFMG 386
Blanc_Spn1      IHKAFVEVNEEGSEAAAATAVNMMKRSLDG-----ETFFADHPFLFLIRDNDSNSILFLG 393
Blanc_Spn6      IHKAFVEVNEEGSEAAAATAVNMMKRSLDG-----ETFFADHPFLFLIRDNDSNSILFLG 393
Bflor_Spn1      VHKAFVEVNEEGSEAAAATAVNMMKRSLDG-----EMFFADHPFLFLIRDNDSNSVLFLG 393
Blanc_Spn2      LHKAFVDVNEEGTEAAAATAGDIVLSGPT------YEFDADRPFLFFIKDNDTNSILFMG 391
Bflor_Spn2      VHKAFVEVNEKGTEAAAATAGLILLSGPT------HEFAADHPFLFFIKDNETNSILFMG 391
Blanc_Spn8      VHKAFVEVYEKGSEAAAATGVIAVAKSGTFYPEPPPVIRADRPFLFLIRDNRNDSILFIG 346
```

```
Bflor_Spn8          VQKAFVEVNEKGSEAAAATGVVIRLMSGNFWLETPPTVRADRPFLFLIRDNRNDSILFMG 346
Bflor_Spn9          LHKAFVEVNEEGSEAAAATAVVMRGRSGNFG----RSFMVNRPFLFFIQHKPTGTILFLG 403
Bflor_Spn10         LHKAVIEVSEEGTDGAAAPAAAITDRS-RRG----FEFRADHPFLFLIRDKRTGSVLFLG 409
                    ::**.: : *:*::.*.*         .          .  ::**:*:: .: . : **:*

A1                  KVVNPTQK------- 394
Blanc_Spn1          RLVRPEGLTT-KDEL 407
Blanc_Spn6          RLVRPEGHST-KDEL 407
Bflor_Spn1          RLVRPEGHTT-KDEL 407
Blanc_Spn2          RLVRPEGTTTRKDEL 406
Bflor_Spn2          RLVRPEGTTTAKDEL 406
Blanc_Spn8          RVTDPTGNK------ 355
Bflor_Spn8          RVADPTGGKE----- 356
Bflor_Spn9          RVTNPNE-------- 410
Bflor_Spn10         RLVDPRN-------- 416
                    ::  *
```

2. HCII sequences

green: positions of non-standard introns

red: positions of conserved introns

turquoise: intron outside conserved serpin domain

```
A1                 ------------------------------------------------------------
Fugu_HCII          MWVISLVCVAYLMVSPSLAGNIDLSSAFS-DPKP----DPRGFEGA--AVDIEAIPLEFH 53
Tetra_HCII         MWVISLVCVAWLMASPSLAETKHPSSPLS-DPKP----DPRGFEGT--EMDIEALPLEFH 53
Gast_HCII          MWVLGAVSVACLLVAPSLAEIKDLGSHFA-DPE------PRGFEVG-GGADMEAVPLEFH 52
Oryz_HCII          MWVIMVIAAASLLAAPCSAGIKDLGTHFS-GPDP----NPRGLQPAG-TEDVEAIPMEFH 54
Dan_HCII           MWLVPVIVVACLLNSPALAGVKDLSSHFS-TLEKEKTVDARGLSPGGENTDMESIPLDFH 59
Hum_HCII           MKHSLNALLIFLIITSAWGGSKGPLDQLEKGGETAQSADPQWEQLNNKNLSMPLLPADFH 60
Chick_HCII         MKFLFPLLALAVIITSTFCGIKDFSDHFE--------SLKDAHTHENGTYNMPDLPLEFH 52
Xenopus_HCII       MKLLHLATLFLLIHSTLGGIKDLQEHFEDTSNGIRPRGSQSQAVEN-------------L 47
Petro_HCII         MFLYGLIFALSVLQWVEGQDQTKTAVDVNKDAQLSFSRYPNKPSDSLMDDTLALELDGFT 60

A1                 --------------------------------------------------------EDPQ 4
Fugu_HCII          KENTVTT--EILFDGFEDEDYID-FDKILS---SDEYFEGDNIDEIATPAPDIDIFAEPS 107
Tetra_HCII         KENTVTK--EIIFDGFEDEDYID-FDKILAEG-SDDYSDGDNIDEIATPAPDIDIFAEPS 109
Gast_HCII          KENTVTN--DLLFDGFEDDDYID-FDKILAAG-SDDYTEGDEIDEIATPAPDIDIFAEPS 108
Oryz_HCII          KANTVTN--ELVFDGFEDEDYID-FDKILAAG-SDDYIEGDEIDEISTPPPDIDIFAEAS 110
Dan_HCII           RENTVTN--DLP-EGQDDEDYVD-FDKILG---EDDYSEGDHIDEISTPAPDLDLFYEPS 112
Hum_HCII           KENTVTN--DWIPEGEEDDDYLD-LEKIFS-------EDDDYIDIVDSLS------VSPT 104
Chick_HCII         RENTITN--DLIPEEEEEEDYLD-LDKILG--------EDDYSDIIDAAP------HIV- 94
Xenopus_HCII       QDDTVTN--DLITEGEEEDDYLD-FDKIFG-------EDGDYIDIIDAAP------EIK- 90
Petro_HCII         DEDSLEDYIDFDKLLNEDDDYPDEIDDINEDGSTGVTVDAEKVGLLHFTLSFSTEIKNLV 120

A1                 GDAAQKTDTSHHDQDHPTFNKITPNLAEFAFSLYRQLAHQSN-STNIFFSPVSIATAFAM 63
Fugu_HCII          DPKIRRARLLRLFQGQSRLQRLNIINARFGFNLYRSLRNTVNQSDNILLAPAGISIAIGM 167
Tetra_HCII         DPKIRRARLLRLFHGQSRLQRLNIVNAHFGFNLYRSLRNTVNQSDNILLAPAGISIAMGM 169
Gast_HCII          DPKIRRARLLRLFHGRSRLQRLNIVNAHFGFNLYRSIRNDVNQSDNILLAPAGISVAMGM 168
Oryz_HCII          DPKIRRARLLRLFHGRSRLQRLNTVNAHFGFNLYRSLRNHVNQSDNILLAPAGISIAMGM 170
Dan_HCII           DPKIRRARLLRLFHGQTRLQRINVVNARFGFRLYRKLRNRLNQTDNILLAPVGISIAMGM 172
Hum_HCII           DSDVSAGNILQLFHGKSRIQRLNILNAKFAFNLYRVLKDQVNTFDNIFIAPVGISTAMGM 164
Chick_HCII         -SEIQQGNILELFQGKTRIQRLNILNANFGFNLYRSVADKANSSDNILMAPVGISTAMAM 153
Xenopus_HCII       DSESQQGNIYELFQGKTRVQRLSIINANFGFNLYRAIKNNTDASDNILLAPVGISTAMAT 150
Petro_HCII         DASFNKKLFLRRFQGKTRIQRLSIVNSDFAFNLYRSVSESTPSGENLLLAPLGISSTLGM 180
                   .         .  :.:. .:::.   : *.* *** : .      *::::* .*: ::.

                                    83c         ----helix D-----
A1                 LSLGTKADTHDEILEGLNFN-----LTEIPEAQIHEGFQELLRTLNQPDSQLQLTTGNGL 118
Fugu_HCII          MSLGTGAGTHDQIYKAMGFSEFVNASHHYDNTTVHKLFRKLTHRLFRRNFGYKLRAVNDV 227
Tetra_HCII         MSLGAGAETQDQIYKAMGFSEFVNASHCYDNTTVHKLFRKLTHRLFRRNFGYNLRAVNDV 229
Gast_HCII          MSLGAGSGTRDQIYGALGFADFVNASHHYDNTTVHKLFRKLTHRLFRRNFGYTLRSVNDV 228
Oryz_HCII          MSLGAGPGTHEQIYEALGFADFVNASHHYDNTTVHKLFRKLTHRLFRRNFGYTLRSVNDV 230
Dan_HCII           MGLGVGPNTQEQLFQTVGFAEFVNASNHYDNSTVHKLFRKLTHRLFRRNFGYTLRSVNDL 232
```

```
Hum_HCII          ISLGLKGETHEQVHSILHFKDFVNASSKYEITTIHNLFRKLTHRLFRRNFGYTLRSVNDL 224
Chick_HCII        ISLGLKGQTQQEVLSVLGFEDFINASAKYELMTVHNLFRKLTHRLFRRNFGYTLRSVNDL 213
Xenopus_HCII      ISLGTKGQALDQVLFTLGFKNFINASSKYEILTLHNVFRKLTHRLFRRNFGYTLRSVNDI 210
Petro_HCII        IALGANGGTHKEIYKALGFESLVDSSSKYNISTVHKLFHRLNHRLFRHNFGYTLKSASAL 240
                  :.**    : .::   : *              :*: *:.* : * : :    * : . :

A1                FLSEGLKLVDKFLEDVKKLYHSEAFTVNFGDTEEAKKQINDYVEKGTQGKIVDLVKELDR 178
Fugu_HCII         YVKKDVAVKDVFRAETKAYYFAEPQSVNFRD-PGFLDKANSRILKLTKGLIRQPLKSIDP 286
Tetra_HCII        YIKKDVAVKDAFRAETKAYYFAEPQSVNFRD-PAFLDKANSRILKLTKGLIRQPLKSIDP 288
Gast_HCII         YVKREVAVKDAFRAETKAFYFAEPQSVDFGD-PAFLDKANSRILKLTKGLIKEPLKSVDP 287
Oryz_HCII         YIKKEVSMKDGFRTQSKNYYFAEPQSVNFRD-PAFLEKANSRIQKLTKGLIKDPLKSVDP 289
Dan_HCII          YVKRNVQIQDSFRADAKTYYFAEPQSVDFAD-PAFLVKANQRIQKITKGLIKEPLKSVDP 291
Hum_HCII          YIQKQFPILLDFKTKVREYYFAEAQIADFSD-PAFISKTNNHIMKLTKGLIKDALENIDP 283
Chick_HCII        YIRKDFSILNDFRNNMKTYYFADAQPADFSD-PNFITKTNERILKLTKGLIKEALVNVNP 272
Xenopus_HCII      YVKKDFVIREPFKNNLKNYYFAEAQMVDFGS-KDFLTKANKRIQQLTKGLIKEALTNVDP 269
Petro_HCII        YLQRRWPLLPSYQQCLRKTYFAEAHTVDFKD-PATVQRINRWVSSATKGTISDAVTNIDP 299
                  ::..   :   :    :  *.:*.  .:* .      : *  : . *:* * : :  .:*

                              192a
A1                DTVFALVNYIFFKGKWERPFEVKDTEEEDFHVDQVTTVKVPMMKRLGMFNIQHCKKLSSW 238
Fugu_HCII         NMVLMLLNYLYFKGTWEQKFPKENTHYRNFRVNEKTSVRVPMMINKGNYLAAADHELECD 346
Tetra_HCII        NMVLMLLNYLYFKGTWEQKFPKESTHYRNFRVNEKTQVRVPMMINRGNYLAAADHDLDCD 348
Gast_HCII         NMVLMLLNYLYFKGAWEQKFPKEMTHYRNFRVNEKTNVRVPMMTNKGNYLAAADHELQCD 347
Oryz_HCII         NMVLMLLNYLYFKGTWEQKFPKEMTYYRNFRITEKTSVRVPMMANKGNYLAAADHELDCD 349
Dan_HCII          NMAVMLLNYLYFKGTWEQKFPKELTHHRQFRVNEKKQVRVLMMQNKGSYLAAADHELNCD 351
Hum_HCII          ATQMMILNCIYFKGSWVNKFPVEMTHNHNFRLNEREVVKVSMMQTKGNFLAANDQELDCD 343
Chick_HCII        TTLMMILNCLYFKGTWENKFPVEMTTKRSFRLNEKQTIKVPMMQTKGNFLAAADPELDCG 332
Xenopus_HCII      ALLMLLVNCIYFKGTWENKFPVEYTQNMNFRLNEKELVKVPMMKTKGNFLVAADPELDCA 329
Petro_HCII        STVFLVINSVYFKGPWEIKFSKHQTSVRSFRLNDKETVKVQMMQTKASFLVTTDHELGCD 359
                     . ::* ::*** *   *  . *   .*:: :   *:* **   . :      .* .

                   241c                                       282b
A1                VLLMKYLGNATAIFFLPD--EGKLQHLENELTHDIITKFLENEDRRSASLHLPKLSITGT 296
Fugu_HCII         ILQLPYTGNISMLIALPRKITG-MRTLEQEISPTVVSKWFKNMTNRTREVVIPRFKLEQS 405
Tetra_HCII        ILQLPYRGNISMLIALPRKITG-MRTLEQDISPTVVSKWLKNMTNRTREVVLPRFKLEQS 407
Gast_HCII         ILQLPYTGEISMLIALPSKING-MRTLEQEISPTVVKKWLKNMTNRTREVAIPRFKLEQN 406
Oryz_HCII         ILQLPYSGNISMLIALPRKISG-MRTLERQISPTVVNKWLKNMTNRTREVLLPRFTLEQS 408
Dan_HCII          ILQLPYAGNISMLIAVPQKLSG-MRSLEQEISPTLVNKWLSNMTNRTREVVFPRFKLEQN 410
Hum_HCII          ILQLEYVGGISMLIVVPHKMSG-MKTLEAQLTPRVVERWQKSMTNRTREVLLPKFKLEKN 402
Chick_HCII        VIQLPFVGNISMLIVLPHKLSG-MKALEKQITPQVVEKWQKSMTNRTREVVLPKFKLEKN 391
Xenopus_HCII      VLQLPYVGNISMLIVLPHKLSG-MKLLEKQISPQVVERWQNIMTNRTREVFLPRFKLEKS 388
Petro_HCII        ILQLAYQGNVSMILAVPHKLKGGLKTLERALSFDLLEKWLQAMTNRTRDVIIPKFNLQQK 419
                  :* : * *  : :: :*    * :: **  ::  :: :: .   .*: .: :*::.:  .

                                                 331c
A1                YDLKSVLGQLGITKVFSNGADLSGVTEEAPLKLSKAVHKAVLTIDEKGTEAAGAMFLEAI 356
Fugu_HCII         YDLIENLQELGLTDMFKDSGDFSEMT-SEKVSMNW-KHQGTITVNEEGTEAAALTQVGFM 463
Tetra_HCII        YDLIENLQKLGLTDLFESSGGFSEMT-SEKVSMNWLKHQGTITVNEEGTEAAALTQVGFM 466
Gast_HCII         YDLIANLKEMGLTDLFQESGDFSAMT-SDKVHMSWLKHQGTITVNEEGTEAAALTQVGFM 465
Oryz_HCII         YDLIQNLKEMGLSDMFQGSGDFSGMT-SERVVMSWLKHQGTITVNEEGTEAASLTQVGFM 467
Dan_HCII          YDLIEHLKEMGMTDIFTEKGDFSPMT-SEKVIINWFKHQGSITVNEEGTEAAAMTHIGFM 469
Hum_HCII          YNLVESLKLMGIRMLFDKNGNMAGIS-DQRIAIDLFKHQGTITVNEEGTQATTVTTVGFM 461
Chick_HCII        YNLIGFLRSMGIEELFSEKGNYCGVS-EEKVSIDRFNHQGTITVNEEGTEAGAITNVGFM 450
Xenopus_HCII      YNLQEVLSNMGVTDLFT-HGDFSGVS-DKNMNIGLFQHQGTITVNEEGTEAAAVTVVGFM 446
Petro_HCII        YNLKNNLKELGVTELFQANADLSGMTGAKDVQVSSFQHQGFIKIDEEGSEAAAVTTVGFT 479
                  *:*   *  :*:  :*   ..:: ::    : :.   *:. :.::*:*::*:    :

A1                PMSIPPEVKFNKPFVFLMIEQNTKSPLFMGKVVNPTQK 394
Fugu_HCII         PLSSQIRFTVDHPFLFLIYEHRTDCLVFIGRVSNPSQS 501
Tetra_HCII        PLSSQIRFTVDHPFLFLIYEHRTDCLVFIGRVVDPSQS 504
Gast_HCII         PLSSQIRFTADRPFLFLIYEHRTDCLVFMGRVVNPSQN 503
Oryz_HCII         PLSSQIRFVVDHPFLFLIYEHRTDCLVFIGRVVNPSQS 505
Dan_HCII          PLSTQTRFIVDRPFLFLIYEHRTGCVVFMGRVVDPSQS 507
Hum_HCII          PLSTQVRFTVDRPFLFLIYEHRTSCLLFMGRVANPSRS 499
Chick_HCII        PLSTQIRFIVDRPFLFLIYEHRTNCLLFMGRVVNPAKP 488
Xenopus_HCII      PLSTQARFVADRPFLFLIYEHRTNCLIFMGRVANPTKS 484
Petro_HCII        PLTSHNRFVADRPFVFIVYEHHTMSVLFLGQVSNPAKN 517
                  *::   ..  ::**:*:: *:.* . :*:*:* :*::.
```

3. Spn_94a sequences

green: positions of non-standard introns

red: positions of conserved introns

```
A1              ----------EDPQGDAAQKTDTSHHDQDHPTFNKITPNLAEFAFSLYRQLAHQSNSZNI 50
Fugu_Spn_94a    ------MTPLLSLLLPGFLLLGLASPAITDGSLEKLTNGNTDFAAKLYQAVASRTD-DNV 53
Tetra_Spn_94a   ------MIPLFSLLLAGFLFLDLVSAQIPDGSVENLASRNVDFAARLYQAVASRTD-DNV 53
Gast_Spn_94a    ------------MAPSLLSLVGLLLLSLCSSEADVLTDSNADFSARLYHVVSSRTD-DNI 47
Oryz_Spn_94a    MVTMAPCTPLLPVILL-LVLLGPASQETINPALVDLTNRNADFGTRMYRAIASRTD-DNI 58
Dan_Spn_94a     -----MKMGFFTLLIEASLLSVSVLGQTTD--VEELAIKNADFATRLYSKIASSSD-DNV 52
                                             .    .:    .:*.  :*  ::  ::  *:

                                           94a
A1              FFSPVSIATAFAMLSLGTKADTHDEILEGLNFN-LTEIPEAQIHEGFQELLRTLNQPDSQ 109
Fugu_Spn_94a    CLSTFALSTALSALLSATSGPTQEQLLQGLGLTGLDAQMLP---ELFQNLRTAIQPGN-I 109
Tetra_Spn_94a   CLSTFALSSVLSALLSATSGPTREQLSQGLALTGLDPQTLP---DLFQNLRTTTQQGNTA 110
Gast_Spn_94a    FLSTFTLTNGLLALVSGTNGPTKDQLLQALSLTGLDSQALP---DLFQTLRTLVLATS-- 102
Oryz_Spn_94a    FLSPFTLSTGLLVLLSATSGSTQEQLFQGLTLTGLDHQSLP---DLFRTLRNVVLRRD-A 117
Dan_Spn_94a     AVSTLGATLALATLAAGAGGATQSELLQGIGVDSMVKDGEQ------ERIQNILQQLRED 106
                 .*..  :  :  *  .: . *:.:: :.: .  :            . :

A1              LQLTTGNGLFLSEGLKLVDKFLEDVKKLYHSEAFTVNFGDTEEAKKQINDYVEKGTQGKI 169
Fugu_Spn_94a    TNLKQAVALLPSHNFEVSASLRELVQTKFGGYMPSLKYTDQAEAISTINRWAQDQTGDQI 169
Tetra_Spn_94a   TCLKQAVAVLPSNNFEVSASFRQLVQTKFGGYIPNVRYSDQAEAISTINRWAQDQTGDKI 170
Gast_Spn_94a    --LKQGVAVLPAVGFQLSASYLDLVQSKFGGNAVNVAYTVPQEATDTINQWAQDQTGDQD 160
Oryz_Spn_94a    ANLQQGAAIFPGENFQLSTAFQDLVQNKFGVKAQKLSYTLPRESEDAINGWAQEQTEGKI 177
Dan_Spn_94a     AAQIPATGLFIKQDVKADDSFSNQVKQYYNADVQNVNYANGQQAKGSINDYVRGRTGEKV 166
                     . .::   ..:      : *:  :     .: :    ::   ** :..  *  :

                                     192a
A1              VDLVKELDRDTVFALVNYIFFKGKWERPFEVKDTEEEDFHVDQVTTVKVPMMKRLGMFNI 229
Fugu_Spn_94a    QQVVTAVDAQTELLLATVSYYKTQFSPLFNASLTQDERFYVNKYVVVMVPMMFRADKYFL 229
Tetra_Spn_94a   QQFVTALDAQTQLLLATVSYYQTQFSPLFNASLTQDERFYVNKYAVVMVPMMFRADKFFL 230
Gast_Spn_94a    QDLMTNWDPNTQLLLATIGSFKAQFTPSFNASLTQDERFYVDNYHVVMVPMMLRADKFFL 220
Oryz_Spn_94a    QELVPTLDPQTQLLLASVASYQTRFNPPFNSSVTLDERFFVDKYHVVMVPMMFRADKYFL 237
Dan_Spn_94a     RDVVENVDPQSMAILISAAFFTGQWLQPFNATFTQEDRFYVNKYNIVQVPMMLRSGKYYL 226
                 :.:   * ::   * .   :  ::   *: . * :: *.*::   * **** * . : :

                                                    282b
A1              QHCKKLSSWVLLMKYLGNATAIFFLPDE-GKLQHLENELTHDIITKFLENEDRRSASLHL 288
Fugu_Spn_94a    AYDRSLKVGVLKLPMGDSMAMLVVLPDEDVDIIVVEEKVTGEKIRGWIRQLKKTKLEVQL 289
Tetra_Spn_94a   AYDPLLKVGVLKLPMSDGTAMLVVLPDEDVDIIDVEEKMTGEKIRAWIRQLKKTKLEVQF 290
Gast_Spn_94a    AYDPSVKVGVLKLPMAGEAAMLVLLPDEGVDISTVEEEITGNKIQSWIRKLKKTKLEVQL 270
Oryz_Spn_94a    AYDRSVKVGVLKLPMADGAAMLVVLPDEDVDITAVEDEVTSENIQAWIRQLKKTKLEVQL 297
Dan_Spn_94a     AYDPTFKVGILKLPCENGIAMLVLLPDEDVDYTYVDESMTGEVFRGWVAKLKKTKLEIQL 296
                 :   ..  :* :    .  : :..****  .   :::.:* : :  :: : .: . .:::

                                         331c
A1              PKLSITGTYDLKSVLGQLGITKVFSNGADLSGVTEEAPLKLSKAVHKAVLTIDEKGTEAA 348
Fugu_Spn_94a    PRFMLEKSYALRDVLQTLNMNKMFQDDADIIEMG-SKGPKLTQVYQKSAVFVGDSRDEVA 348
Tetra_Spn_94a   PRFLLEKSYMMGDFLQTLNVTMVFQDGAEIREMG-AKGPRLTQVYQTSALSVRDSSEEVM 349
Gast_Spn_94a    PRFLLERSYFLRDVLQTLDITRVFQDDADLSTIGGGAGLKLTQVFHKSVLSVDESSDDIT 340
Oryz_Spn_94a    PRFTLESSYSLKDTLQTLHITQVFEDDADISNMGGAKGTKLTQVYHKSVISVDEATDDGI 357
Dan_Spn_94a     PRFSLKQSNSLSVSLPSLGVKEIFGSTANLTGISSSEGLKLSEVVQKVAVDVDESGGSLA 346
                *:: :  :  :   *  * :. :* . *::  :      :*::. :. .: : :   .

A1              GAMFLEAIPMSIPPEVKFNKPFVFLMIEQNTKSPLFMGKVVNPTQK-- 394
Fugu_Spn_94a    TAGGASTF-SYPPPRLTINRPFLFIIYHQTIGSVLFMGRVTNPIA--- 392
Tetra_Spn_94a   TGGGASMF-SDPPPRLTINRPFVFLIYHQMSGIVLLIGRVSDPTLQ-- 394
Gast_Spn_94a    AEGGASSS-SSLPPRLTVNRPFVFVIYQETSGAVLSMGRVVNPSRK-- 385
Oryz_Spn_94a    Q-GRDAAF-ATPPPRLTINRPFIFIIFQQTTGSLLFMGRVTNPLKN-- 401
Dan_Spn_94a     EASGNLFM-NPLPPRLTFNRPFIFVVYHEVTKCILYIGRVVDPTCYNT 393
                            **.:..*:**:*:: .:     * :*:* :*
```

4. Spn_215c sequences

green: positions of non-standard introns

red: positions of conserved introns

```
A1                -----------------EDPQG-DAAQKTD--TSHHDQDHPTFNKITPNLAEFAFSLYRQ 40
Tetra_Spn_215c    MSATLRLCVLCLMLLVARGHQG-DGAEKLEGQQSSAANSSAGVPLLTAANREFAFRLYRS 59
Fugu_Spn_215c     MNATRCVWILSIIICVARGHVGNDIGQNQKEQDTSADNSTESLSLVTAANREFAFRLYRS 60
                                   ..  * * .:: .   :   :.   .  :*.   **** ***.

A1                LAHQSNS--TNIFFSPVSIATAFAMLSLGTKADTHDEILEGLNFNLTEIPEAQIHEGFQE 98
Tetra_Spn_215c    LAAQPDSRGKNVFFSPLSVSVALAALAVGARGETHQQLFRGLGLSNTSLSQAQVDQAFQS 119
Fugu_Spn_215c     LAANPDSQGKNIFFSPVSVSVALAALAVGARGETHRQLFRGLAFNSTWLSQTDVDQAFQS 120
                  ** :.:*  .*:****:*::.*:* *::*::.:** :::.** :. * :.:::::.:**.

A1                LLRTLNQPDSQLQLTTGNGLFLSEGLKLVDKFLEDVKKLYHSEAFTVNFGDTEEAKKQIN 158
Tetra_Spn_215c    LFEQTRRTSSQVTR-EGTAVFVDHLFKAQPGFLHTLKQSYFADGFAVDFSKSSESTDTIN 178
Fugu_Spn_215c     LFEKTKKASNEVTS-EGTAVFMDNLFKPQPEFLDTLKKSYFADGFNVDFTKSSESANTIN 179
                  *:.  .:...::    *..:*:.. :*    **. :*: *.::.* *:* .:.*: . **

                                                  192a                  215c
A1                DYVEKGTQGKIVDLVKELDRDTVFALVNYIFFKGKWERPFEVKDTEEEDFHVDQVTTVKV 218
Tetra_Spn_215c    KYVKEKTSGKIDKLVKDLDPSTVMYLISYIYFKGKWESPFDPDLTQEDVFTVDEETKVPV 238
Fugu_Spn_215c     KYVEEKTSGKIDKLVESLDPTTVMYLISYIYFKGKWETPFDPDLTKEDLFMVDEKTKVPV 239
                  .**:: *.*** .**:.**  **: *:.**:****** **: . *:*: * **: *.* *

A1                PMMKRLGMFNIQHCKKLSSWVLLMKYLGNATAIFFLPDEGKLQHLENELTHDIITKFLEN 278
Tetra_Spn_215c    QMMNLERRFETYHDQTVNTSVLRLPFNSSHSMLLLLPEH--MAQLEQALSPAHISKWLKW 296
Fugu_Spn_215c     QMMNIEKRFETYRDQMFNTSVLHLPFNSSHSMLLLLPDD--MSKLENAISAAHVTKWLKW 297
                   **:    *:  : : ..: ** : : .. : :::**:.  : :**: ::   ::*:*:

                    282b                                             331c
A1                EDRRSASLHLPKLSITGTYDLKSVLGQLGITKVFSNGADLSGVTEEAPLKLSKAVHKAVL 338
Tetra_Spn_215c    MKSRTFNVYVPKFSIKTSASLKDVLTEMGMADMFGDRADLTGISEGGRLSVSEVVHQATL 356
Fugu_Spn_215c     MKYRKYSVYIPKFSIKTSYSLKTVLTEMGMVDMFGDRADLSGIAEGQQLAVSEVVHQATL 357
                   . *. .:::**:**. : .** ** ::*:..:*.: ***:*::*   * :*:.**:*.*

A1                TIDEKGTEAAGAMFLEAIPMSIP--PEVKFNKPFVFLMIEQNTKSPLFMGKVVNPTQK 394
Tetra_Spn_215c    DVDEAGATAAAATGIGITLFSFHHVPVLKFDRPFMVIITEHSTESILFLGKITNPKI- 413
Fugu_Spn_215c     DVDEAGATAAAATGIAITLFSYNYVPVLKFNRPFMVIITDHSSDNILFMGKITNPNI- 414
                   :** *: **.*  :    :*    * :**::**:.:: ::.:.. **:**:.**.
```

5. AT sequences

red: positions of conserved introns

pink: intron position possibly lost in tetrapods

```
A1                -----------------------------------------EDPQGDAAQKTDTSHH--- 16
Fugu_AT           ---------------MPASDWLLLLASLHVVS-ADVLDICGAKPRDLALEPRCIYRS--- 41
Gast_AT           ---------------MKAADWLLVLVSLPLVSFAFDSDICSAKPKNIPLEPRCIYRS--- 42
Oryz_AT           ---------------MRATHWLLVLAFLPSIVLAQHVDICNAKPKDLPLEPRCSYRS--- 42
Dan_AT            ---------------MKLLACMWALWAFALCSIHATKDICNAKPKDLPLEPMCIYRN--- 42
Hum_AT            MYSNVIGTVTSGKRKVYLLSLLLIGFWDCVTCHGSPVDICTAKPRDIPMNPMCIYRS--- 57
Xenopus_AT        ---------------MYLLSLLLLSLLGSGYLQSQNADICLAKPKDIPLTPMCVYRKTLE 45
                                                            .*:. .       :

A1                -----------------------DQDHPTFNKITPNLAEFAFSLYRQLAHQ-SNSTNIFF 52
Fugu_AT           ---PDPEAPEP---LTTHP--VPGSTNPRVWELSKANARFAMSLYKQVASSRGPESNIFM 93
Gast_AT           ---PDPE-PEPGQGPTPQPETIPESTNPRVWELSKANSRFALSLYKHLALDKAPEANIFM 98
```

```
Oryz_AT         ---PDLETPED---HTPEPKVVPESTNPRVWELSKANSHFALSFYKHLAVSRKTEENIFM 96
Dan_AT          ---PDEIQPNK------EPENIPVGTNPRVWELSKANSRFALSLFKQLAEGKSNDENIFL 93
Hum_AT          ---PEKKATED----EGSEQKIPEATNRRVWELSKANSRFATTFYQHLADSKNDNDNIFL 110
Xenopus_AT      AVEAEEKEKEP----AQQEQKVPESTNPRVYELSQANAKFAIAFYKNLADSKQNTENIFM 101
                                          :  . :::   :.** ::::::*       ***:

                        78c          -----helix D----
A1              SPVSIATAFAMLSLGTKADTHDEILEGLNFN-LTEIPEAQIHEGFQELLRTLN-QPDSQL 110
Fugu_AT         SPISISTAFAMTKLGACNQTLEQLMRVFEFDTIKEKTSDQVHFFFAKLNCRLYRKKDKSN 153
Gast_AT         SPISISTAFAMTKLGACNQTLRQIMEVFEFNGIKEKTSDQVHFFFAKLNCRLYRKKDKTT 158
Oryz_AT         SPISISTAFAMTKLGACNRTLEQIMKVFQFDTIKEKTSDQVHFFFAKLNCRLYRKKDETT 156
Dan_AT          SPISISTAFAMTKLGACNTTLEQLMKVFQFDTIKEKTSDQVHFFFAKLNCRLYRKKHETT 153
Hum_AT          SPLSISTAFAMTKLGACNDTLQQLMEVFKFDTISEKTSDQIHFFFAKLNCRLYRKANKSS 170
Xenopus_AT      SPLSISQAFTMAKLGACNNTLKELMEVFYFDTISERASDQIHYFFAKLNCRLFRKANKSS 161
                **:**: **:* .**:   *  :::. : *: :.*  .. *:*  * :*   *  : ..

                                                    148c
A1              QLTTGNGLFLSEGLKLVDKFLEDVKKLYHSEAFTVNFGD-TEEAKKQINDYVEKGTQGKI 169
Fugu_AT         ELVSANRLFGDKSLAFDQTYQNISETVYGAKLLPLDFKDDPEKARVTINNWISNKTENLI 213
Gast_AT         ELISANRLFGDKSLVFNATYQNISEAVYGAKLLPLNFKENPEKARVTINDWIANKTENLI 218
Oryz_AT         ELVSANRLFGEKSLFFNETFQNITEMVYGAKLLPLNFKMYPEQARITINDWISNKTENRI 216
Dan_AT          ELISANRLFGDKSTTFNETFQHISETVYGAKLMPLDFKEKPEASRITINEWIANKTENRI 213
Hum_AT          KLVSANRLFGDKSLTFNETYQDISELVYGAKLQPLDFKENAEQSRAAINKWVSNKTEGRI 230
Xenopus_AT      ELVSVNRLFGEKSLTFNETYQDISELVYGAKLLPLNFKEKPELSREIINNWVSDKTEKRI 221
                :* : * ** .:.  :  .: .  : :* ::  .::*   .* ::  **.:: . *:  *

                      191c
A1              VDLVK--ELDRDTVFALVNYIFFKGKWERPFEVKDTEEEDFHVDQVTTVKVPMMKRLGMF 227
Fugu_AT         QDTLPPGVLDSNTVLVLVNTIYFKGHWKNKFDKDNVYVSEFHSSQTRSCPVNMMYQEARF 273
Gast_AT         QDTLPEGALDSNTILVLVNTIYFKGQWKNKFDKDNVYVADFQVSAARTCSVKMMYQETKF 278
Oryz_AT         KDTLPSGVLDSNTVLVLVNTIYFKGQWKKKFDKKAVFSSDFQVSSSHNCQVNMMFQESKF 276
Dan_AT          KDTLPEGSIDTNTILVLVNAIYFKGQWKNKFDKQNVMKLDFHVSPTHKCPVPMMYQEKKF 273
Hum_AT          TDVIPSEAINELTVLVLVNTIYFKGLWKSKFSPENTRKELFYKADGESCSASMMYQEGKF 290
Xenopus_AT      TDVIPVGVITPDTVLVLINAIYFKGLWKSKFNSENTKMEQFYPDESNHCLAATMYQEGIF 281
                 * :    :   *::.*:* *:*** *:  *. . .    *         .  * :   *

                                                   262c
A1              NIQHCKKLSSWVLLMKYLG-NATAIFFLPDEGK-LQHLENELTHDIITKFLENEDRRSAS 285
Fugu_AT         RYKHFPEDQVQLLEMPYRGDDITMVIILPSQGTALSQVEEVLDLKKLSAWLDQMKETTVS 333
Gast_AT         RYKNFPDDQVELLEMPYRGDDITMVIILPMKNTPLSKVEEGLDLNKLTSWLDGMKETTVA 338
Oryz_AT         KYKYFPDDEVQVLEMPYIGDDITMVIILPSRNTPLNQVEESLNLKKLNNWLDQMTETSVS 336
Dan_AT          QYAKIPEDKVKILELPYNGGDITMVLILPIEGATLSEVVANMNLKKLVGWLHAMKETTVA 333
Hum_AT          RYRRVA-EGTQVLELPFKGDDITMVLILPKPEKSLAKVEKELTPEVLQEWLDELEEMMLV 349
Xenopus_AT      RYSSFKDDGVQVLELPYKGDDITMVLVLPSPETPLMKVEQNLTLEKLGNWLQKSRELQLS 341
                .          :* : : * : * ::.**     * .:   :  . :  :*.   .

                                                  320a                    339c
A1              LHLPKLSITGTYDLKSVLGQLGITKVFS-NGADLSGVTEEAP---LKLSKAVHKAVLTID 341
Fugu_AT         VHVPRFRVEDSFSLKEKLQLLGLTDLFDPNKASLPGMLEDGV-EGLHISDAYHKAFLEVN 392
Gast_AT         VHVPRFRVEDSFSVKEKLQAMGLTDLFSSERASLPGMLEDGS-EGLHISDAFHKAFLEVN 397
Oryz_AT         VSIPRFKVEDKFNLKEVLQEIGLTDLFSAENASLPGILEDDS-DGLFISDAYHKAFLEVN 395
Dan_AT          VQIPRFRVEDSFSLKEQLTKMGLEDLFSPANASLPGMVADAEGPNLFISDAYHKAFLEVN 393
Hum_AT          VHMPRFRIEDGFSLKEQLQDMGLVDLFSPEKSKLPGIVAEGR-DDLYVSDAFHKAFLEVN 408
Xenopus_AT      VYLPRFRVEDSFSVKEKLQQMGLVDLFDPNSAKLPGIVAGGR-TDLYVSDAFHKAFLEVN 400
                : :*:: : . :.:*. *  :*: .:*.   :.*.*:         * :*.* ***.* ::

A1              EKGTEAAGAMFLEAIPMSIPP---EVKFNKPFVFLMIEQNTKSPLFMGKVVNPTQK- 394
Fugu_AT         EEGSEAAAATAAVATGRSINLNREIFQANRPFLLLIREASINTLLFIARVAEPCDR- 448
Gast_AT         EEGSEAAAATAVVAVGRSLNLRRPVFMANRPFLLLIRESTINSLLFTARVADPCDQ- 453
Oryz_AT         EEGSEAAAATVVLAVGRSFNPYREVFQADRPFLLFIRESTINTLLFSGRVVNPCDQ- 451
Dan_AT          EEGSEASAATAVVATGRSLNIFREQFVADRPFLLFIRESSINALIFTGRVANPCRSS 450
Hum_AT          EEGSEAAASTAVVIAGRSLNPNRVTFKANRPFLVFIREVPLNTIIFMGRVANPCVK- 464
Xenopus_AT      EEGSEAAASTAVILTGRSLNLNRITFRANRPFLVFIREVAINSVLFMGRVANPCTE- 456
                *:*:**:.:        *:       .  ::**:.:: *   :: :* .:*.:*
```
