# Supplementary material for: Multiple gains of spliceosomal introns in a superfamily of vertebrate protease inhibitor genes
Source: BMC Evol Biol. 2009 Aug 22;9:208. doi: 10.1186/1471-2148-9-208 (PMC2746811; doi:10.1186/1471-2148-9-208)
Supplement: Additional file 2 — List of serpin genes analyzed in this study and their accession numbers. Table listing accession numbers of genes, cDNAs and ESTs investigated in this study. [file 1471-2148-9-208-S2.pdf]

Additional file 2. List of *serpin* genes analyzed in this study and their accession numbers.

**Species**	**Gene (group)**	**Accession; source of data***	**cDNA/EST accession**	**Note**
B. lanceolatum	*Blanc_Spn1* (L1)	AJ889984 (1)	AJ548509 (1)	
B. floridae	*Bflor_Spn1* (L1)	estExt_fgenesh2_pg.C_4600026 [Brafl1:130749] (2)	-	manually refined
B. lanceolatum	*Blanc_Spn2* (L1)	AM180517 (1)	AM114535 (1)	
B. floridae	*Bflor_Spn2* (L1)	fgenesh2_pg.scaffold_1013000002 [Brafl1:112013] (2)	-	manually refined
B. lanceolatum	*Blanc_Spn6* (L1)	AJ889983 (1)	-	Blanc_Spn1 allele?
B. lanceolatum	*Blanc_Spn8* (L2)	FM242707 (1)	-	gene complete?
B. floridae	*Bflor_Spn8* (L2)	fgenesh2_pg.scaffold_11000109 [Brafl1:66648] (2)	-	gene complete?
B. floridae	*Bflor_Spn9* (L3)	estExt_fgenesh2_pg.C_6170006 [Brafl1:131993] (2)	-	manually refined
B. floridae	*Bflor_Spn10* (L3)	fgenesh2_pg.scaffold_11000112 [Brafl1:66651] (2)	-	
L. fluviatilis	*Angiotensinogen* (V2)	FM955146 (1)	FM954978 (1)	
P. marinus	*Angiotensinogen* (V2)	PMAR3:Contig1818:3231:6123:1 (3)	EG022479 (1), DY799969 (1)	gene incomplete
H. sapiens	*Angiotensinogen* (V2)	NG_008836 (1)	K02215 (1)	
G. gallus	*Angiotensinogen* (V2)	ENSGALG00000011117 (4)	BU142914 (1)	
D. rerio	*Angiotensinogen* (V2)	ENSDARG00000016412 (4)	AY049731 (1)	
O. latipes	*Angiotensinogen* (V2)	ENSORLG00000005554 (4)	-	
G. aculeatus	*Angiotensinogen* (V2)	ENSGACG00000009742 (4)	BT026720 (1)	
T. rubripes	*Angiotensinogen* (V2)	ENSTRUG00000010811 (4) (Q6IMN9_FUGRU)	-	manually refined
P. marinus	*HCII* (V2)	PMAR3:Contig1544:23668:28929:1 (3)	-	
H. sapiens	*HCII* (V2)	M58600 (1)	X03498 (1), NM_000185 (1)	

G. gallus	*HCII* (V2)	NC_006102 (1)	AF061728 (1)	
X. tropicalis	*HCII* (V2)	ENSXETG00000022417 (4)	DN034515[a] (1)	
D. rerio	*HCII* (V2)	ENSDARG00000021208 (4)	NM_182880 (1)	
O. latipes	*HCII* (V2)	ENSORLG00000002247 (4)	AV669079 (1), AU179051 (1), BJ905316 (1)	
G. aculeatus	*HCII* (V2)	ENSGACG00000004263 (4)	-	
T. nigroviridis	*HCII* (V2)	ENSTNIG00000015390 (4)	-	
T. rubripes	*HCII* (V2)	ENSTRUG00000013053 (4)	-	
D. rerio	*Spn_94a* (V2)	ENSDARG00000005924 (4)	BC134976 (1)	
O. latipes	*Spn_94a* (V2)	ENSORLG00000018182 (4)	AM155792 (1), DK000009 (1)	
G. aculeatus	*Spn_94a* (V2)	ENSGACG00000013654 (4)	DT996398 (1), DW594658 (1)	manually refined
T. nigroviridis	*Spn_94a* (V2)	ENSTNIG00000004806 (4)	-	
T. rubripes	*Spn_94a* (V2)	ENSTRUG00000016175 (4)	BU805220 (1)	
T. nigroviridis	*Spn_215c* (V2)	ENSTNIG00000004551 (4)	CR635295 (1)	
T. rubripes	*Spn_215c* (V2)	ENSTRUG00000001717 (4)	-	
L. fluviatilis	*Lfl_SpnV4_1* (V4)	FM991712	FM991711	
X. tropicalis	*AT* (V5)	ENSXETG00000020033 (4)	NM_001086610[b] (1)	
H. sapiens	*AT* (V5)	AF386078 (1)	NM_000488 (1)	
D. rerio	*AT* (V5)	ENSDARG00000042684 (4)	AF515272 (1)	
O. latipes	*AT* (V5)	ENSORLG00000014069 (4)	AV668352 (1), BJ706706 (1), BJ912884 (1)	
G. aculeatus	*AT* (V5)	ENSGACG00000013542 (4)	CD507321 (1), DN671625 (1), DT976142 (1)	
T. rubripes	*AT* (V5)	ENSTRUG00000010570 (4) (Q9W648_FUGRU)	-	
G. gallus	*HSP47* (V6)	ENSGALG00000011214 (4)	NM_205291 (1)	

X. tropicalis	*HSP47* (V6)	ENSXETG00000010725 (4)	BC124969[b]	
H. sapiens	*HSP47* (V6)	NC_000011 (1)	NM_001235 (1)	
D. rerio	*HSP47_1* (V6)	ENSDARG00000019949 (4)	BC071301 (1)	
D. rerio	*HSP47_2* (V6)	ENSDARG00000009638 (4)	CO933773 (1), EV760193 (1)	
D. rerio	*HSP47_3* (V6)	ENSDARG00000036445 (4)	BC153557 (1)	
O. latipes	*HSP47_1* (V6)	ENSORLG00000014312 (4)	DK141963 (1), DK132634 (1), AM306105 (1)	
G. aculeatus	*HSP47_1* (V6)	ENSGACG00000006375 (4)	BT027709 (1)	
G. aculeatus	*HSP47_2* (V6)	ENSGACG00000020152 (4)	DN700149 (1)	
T. rubripes	*HSP47_1* (V6)	ENSTRUG00000001559 (4)	-	
P. marinus	*HSP47* (V6)	PMAR3:Contig35119:6440:12437:1 (3)	-	

*The numbers given in brackets refer to source of the data given below:

(1): GenBank

(2): http://genome.jgi-psf.org/

(3): http://pre.ensembl.org/Petromyzon_marinus

(4) http://www.ensembl.org/index.html

[a]: A complete HCII cDNA is known from *X. laevis* (BC049293 (1).

[b]: cDNA from *X. laevis*
