# Supplementary material for: Multiple gains of spliceosomal introns in a superfamily of vertebrate protease inhibitor genes
Source: BMC Evol Biol. 2009 Aug 22;9:208. doi: 10.1186/1471-2148-9-208 (PMC2746811; doi:10.1186/1471-2148-9-208)

Additional file 3. Chromosomal gene order reveals orthology of *Spn_94a* genes. Ray-finned fishes contain multiple *serpins* depicting the standard exon-intron pattern of group V2. With the exception of *Danio rerio*, all fishes investigated share a gene with an extra intron at position 94a (indicated by a plus sign). Chromosomal gene order corroborates that these genes, dubbed *Spn_94a*, are orthologues. Intron gain hence took place after divergence of the *Danio rerio* lineage.

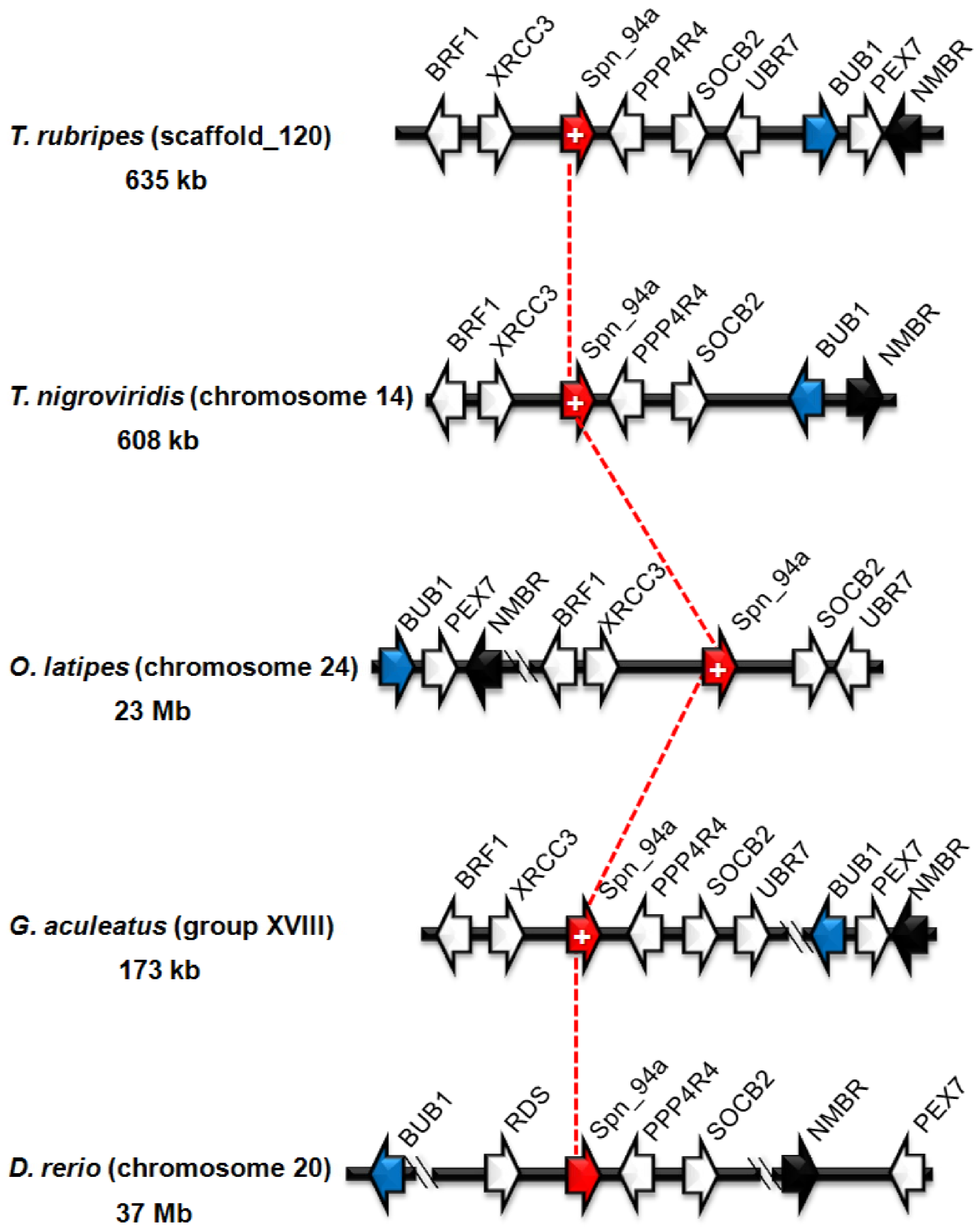

Supplement: Additional file 3 — Chromosomal gene order reveals orthology of Spn_94a genes. Figure showing chromosomal synteny of Spn_94a genes. [file 1471-2148-9-208-S3.pdf]
