# Supplementary material for: Multiple gains of spliceosomal introns in a superfamily of vertebrate protease inhibitor genes
Source: BMC Evol Biol. 2009 Aug 22;9:208. doi: 10.1186/1471-2148-9-208 (PMC2746811; doi:10.1186/1471-2148-9-208)
Supplement: Additional file 4 — Sequences of non-canonical introns from vertebrate serpin genes. Figure depicting the sequences of non-standard introns. [file 1471-2148-9-208-S4.pdf]

Additional file 4. Sequences of non-canonical introns from vertebrate *serpin* genes.

Only introns mapping to the serpin scaffold are considered. The numbering of intron positions refers to the human α_1-antitrypsin sequence. GT-AG splice signals are marked in red.

1. Introns of *angiotensinogen* genes mapping to position 77c

>Fugu_Ang_77c

GTATTATATTCGTGCATGAATACCAGTGTTACACCCACATGACACCCAGCTGAAACCACATCGTCTGCTCTGT**AG**

>Gast_Ang_77c

GTCTCTGATTTTGATTTGTTCTTATGTGTATAGATGAACCTGTTGATCCTTTGCTATTGCGAAAGAGGGACGTGA

CAATCAGGTCTTTTTTCGTTGACTCAGAGATGTTGATTTTGACCGAATCAAACCTTTCCTCGC**AG**

>Oryz_Ang_77c

GTACTTGATTTAGCTTTTTTTTCCTCACAATAAAGATCTTTTTTAAGAGTTTACGTTACAAAAGATTGTTTTTCC

CTTTT**AG**

2. Introns of *angiotensinogen* genes mapping to position 233c

>Fugu_Ang_233c

GTGAATCAGGCTCAAAGATGCTTTCAGCAGGAAGATTCGGGCATTTTGGCTATACCCACTTGCTGTCCTCTTCTA

TCA**AG**

>Gast_Ang_233c

GTACATTTGAATTCAAAAATATCGTTTGTCGCCACATATTTTCCGCTGTAAAATATGAAACTGACTCGCATGAGT

GTTCAAATATAATGGGTGTTACTTTGTCCCTGTGA**AG**

>Oryz_Ang_233c

GTAAGAAGCAGTACAAGAAAGTTAGTGGCTGCTTTTATGCAGGAATTCAACTGACATGTTTTTGGTTTGCCAACC
TGC**AG**

3. Introns of *HCII* genes mapping to position 241c

>Fugu_HCII_241c

GTGGGAAGATTCACATTTGGCATTCTAACGCTTGACTGGATGGGGACTGATGGGCCTTAACGTTCTTATTTTC**AG**

>Tetra_HCII_241c

GTGGGAGGGCTCGCATTTGGCCTTTAACACTCTACCGGAAAAACACACACTGAGCGTCTCTCATCTCC**AG**

>Gast_HCII_241c

GTGGGTGGGCGCTCACTGACCTCCGGAGGTCCCTGAAGACAGACGGCACACAGGTCATTAAAGCATATGTTCTTG
TTTGC**AG**

>Oryz_HCII_241c

GTGGGTGCTCGGCAGCTCTTGTTTGGTGGAGGTGCTCTCTAATCCACAAACCGCTTGGTGACTCGACTTAATGAA
GATGTAACCGTTCTTCCGCGC**AG**

4. Introns of *HSP47_1* genes mapping to position 36b

>Fugu_HSP47_1_36b

GTACCTCCAAAAGCTATTATACCACCTCACTATACAACTAGAAGACGCCTACCTAACTGCAGCTTTTAGTTCAGC
CAAATACTTTCCACTTATTTCAGTTGCCTGCTACATTAGTGTTCCACTACATATGAAGAGAAATAGTGCACTCAT
AATAAAATAATATACACTATCGATGC**AG**

>Gast_HSP47_1_36b

GTCCGACATCTATACATTACTGTGGTTACGATTAATTGTCCTTTAAATGGTGCACTTTTTTCTAAAGCATTATTT

TTTTGTTATATCCCCGTTGGAAAAACACAAGCAAATAAATAACCTTTCTTGTGATTTTCAATTC**AG**

>Oryz_HSP47_1_36b

GTTAGTAATCATTACAAAACACTACAAGGTCATGGTCTGACCTAAATGTGCTGTTTCATCGTTGTAGATGAGTAA

ATTACTGTGATTCTCTTTTCTCC**AG**

5. Introns of *HSP47_1* genes mapping to position 102c

>Fugu_HSP47_1_102c

GTAACGTACTATCTATAAAGTCCAAATCTTAAGGACAGGGTTCTCGTCTATATGGTGCTAAATGTGTCCTGAATT

TTATGCTCCCC**AG**

>Gast_HSP47_1_102c

GTACTTGGAACGATATTTAAAGACCGTATGTGGGACCTCACTGTAATGAGAGGACCCAGCCCTAAGCAGCCCTGA

TATCAGCAGTGCTTCCATTCACACCTGTCATACGTTGCTCCTTGCC**AG**

>Oryz_HSP47_1_102c

GTAAGGAGCTAAAACATCTGGGCTTCTTCAAACAACACTCTGAGTTTATTGGAAAATGTTCACACTTCATCTGTT

GTCCCCTCCCCTCCCCCCCT**AG**

6. Introns of *Spn_94a* genes mapping to position 94a

>Fugu_Spn_94a_94a

GTACAACAGGATTATGGAATGGACTATTTTCTGATCTTAAATTGTGATGTGGCGATTTTTGCTTTC**AG**

>Tetra_Spn_94a_94a

GTATGATGGGATTCGGGGCTGAACTGTTTGCTATGTCTAAACTGTGCGGTGACAGCTACTGTTTTC**AG**

>Gast_Spn94a_94a

GTAGGTCAGGGGTCAGAAGTGAGGGCACCGTCAGTGTGTTGACTGGTAGCTGTGAGAGTCTCCACTGTTTTT**AG**

>Oryz_Spn_94a_94a

GTAAGTCAGGTGTTGTGTCTTCTTCAACACAATAGCCCAAGTTCAGGAGCCAGAGGAGCTGGTGAGATGCCAGAA

TGTGGAGACATAAAGCTGTTCTTCTGGTCTTCTC**AG**

7. Introns of *Spn_215c* genes mapping to position 215c

>Fugu_Spn_215c_215c

GTTTGATGAAGTCCACAGTCTTCGGCAAAACCTTCAACCGAATCTCATTTACAAACATGAAACTTTGAATTTTC**A**

G

>Tetra_Spn_215c_215c

GTCTGCAGGCTTCTGCCACACCCTCAGCTCCAGGCTCAGACCTTCAAGCTGAAACTGTCCATTTCC**AG**
